# Prevalence and future estimates of frailty and pre-frailty in a population-based sample of people 70 years and older in Norway: the HUNT study

**DOI:** 10.1007/s40520-024-02839-y

**Published:** 2024-09-10

**Authors:** Ingebjørg Lavrantsdatter Kyrdalen, Bjørn Heine Strand, Geir Selbæk, Pernille Thingstad, Heidi Ormstad, Emiel O. Hoogendijk, Håvard Kjesbu Skjellegrind, Gro Gujord Tangen

**Affiliations:** 1 The Norwegian National Centre for Ageing and Health, Vestfold Hospital Trust, Tønsberg, Norway; 2 Institute of Clinical Medicine, Faculty of Medicine, University of Oslo, Oslo, Norway; 3 Norwegian Institute of Public Health, Oslo, Norway; 4 Department of Geriatric Medicine, Oslo University Hospital, Oslo, Norway; 5 Trondheim Municipality, Trondheim, Norway; 6 Department of Public Health and Nursing, Faculty of Medicine and Health Sciences, HUNT Research Centre, Norwegian University of Science and Technology, Levanger, Norway; 7 University of South-Eastern Norway, Drammen, Norway; 8 Department of Epidemiology & Data Science and Department of General Practice, Amsterdam UMC – location VU University Medical Center, Amsterdam, The Netherlands; 9 Department of Rehabilitation Science and Health Technology, Oslo Metropolitan University, Oslo, Norway; 10 Levanger Hospital, Nord-Trøndelag Hospital Trust, Levanger, Norway

**Keywords:** Frailty, Pre-frailty, Prevalence of frailty, Projections of future frailty, Physical frailty, Frailty index

## Abstract

**Background:**

Frailty in older people is a rising global health concern; therefore, monitoring prevalence estimates and presenting projections of future frailty are important for healthcare planning.

**Aim:**

To present current prevalence estimates of frailty and pre-frailty and future projections according to both dominant frailty models in a large population-based observational study including adults ≥ 70 years in Norway.

**Methods:**

In this population-based observational study, we included 9956 participants from the HUNT4 70 + study, conducting assessments at field stations, homes, and nursing homes. Frailty was assessed using Fried criteria and a 35-item frailty index (HUNT4-FI). Inverse probability weighting and calibration using post-stratification weights and aggregated register data for Norway according to age, sex, and education ensured representativeness, and population projection models were used to estimate future prevalence.

**Results:**

According to Fried criteria, the current prevalence rates of frailty and pre-frailty in people ≥ 70 years were 11.7% and 41.7%, respectively, and for HUNT4-FI 36.0% and 33.0%, respectively. Compared to previous European estimates we identified higher overall frailty prevalence, but lower prevalence in younger age groups. Projections suggest the number of Norwegian older adults living with frailty will close to double by 2040.

**Conclusion:**

Frailty in older people in Norway is more prevalent than previous European estimates, emphasising the imperative for effective interventions aimed to delay and postpone frailty and ensure healthcare system sustainability in an ageing population. Future planning should consider the great heterogeneity in health and functioning within the 70 + population.

**Supplementary Information:**

The online version contains supplementary material available at 10.1007/s40520-024-02839-y.

## Introduction

Frailty is a multisystem and dynamic clinical condition that affects one’s ability to respond to stressors and increases the risk of functional dependency, hospitalisation and death [1]. Frailty prevalence rises with age, and as the world’s population ages, frailty as a global health concern represents a significant challenge to health systems and societies [1]. Monitoring frailty prevalence is especially important due to its link to greater health-care costs [1]. Frailty surveys provide insight into population health and may help us understand the diversity of ageing [2].

There are two dominant models for defining frailty. One is the physical frailty model, in which frailty is understood to be a distinct high-risk state linked to multisystemic dysregulation [3], frequently measured using Fried criteria [4]. The second model is based on the accumulation of age-related deficits, often called the deficit accumulation model, measured using a frailty index (FI) [5]. In the trajectory from healthy ageing to frailty, pre-frailty is a potentially reversible risk-state. Pre-frailty predisposes to adverse outcomes regarding health and social care as well as progression to frailty [6].

According to a systematic review of studies including community-dwelling people ≥ 50 years, the estimated global prevalence rates of physical frailty and pre-frailty were 12% and 46%, respectively, whereas the corresponding prevalence rates according to FI were 24% and 49% [7]. Regardless of operationalisation, Europe showed the lowest prevalence of frailty among the continents, with 8% using physical frailty criteria and 22% using FI. However, studies included in the review reported widely varying frailty prevalence, data were heterogeneous and only a few studies reported representative data on both frailty models [7].

Previous Nordic studies have reported prevalence rates ranging from 1.6 to 8.4% with Fried criteria [8–10] and from 17.5 to 30.2% with FI [10, 11]. The generalisability of the results of these population-based studies is limited because they excluded individuals with severe functional limitations. As far as we know, there are no nationally representative prevalence studies in Nordic countries that include the oldest age groups and use both frailty models.

To provide valid, updated estimates of the prevalence of frailty, there is a need both globally and for Nordic countries to conduct suitably powered studies applying both frailty models, including all individuals in a geographic area [7], also those not able to attend test stations. For the estimations to be useful to health authorities, both current prevalence numbers and projections of future frailty are necessary. According to the divergent estimates dependent on the choice of frailty model, using both the Fried criteria and FI in the same population facilitates the interpretation of our estimates across different study populations. This wide-ranging approach is also critical for expanding the present knowledge about prevalence of frailty and pre-frailty in Europe to prepare for the near future.

The aim of this paper is to present current prevalence estimates of frailty and pre-frailty according to both dominant frailty models stratified by age groups and sex from a large population-based study in Norway that included both home-dwelling older adults and nursing home residents ≥ 70 years. Furthermore, we will forecast future frailty prevalence for years 2030 and 2040, showing the estimated proportion of the Norwegian population we expect to be living with frailty and pre-frailty.

## Methods

### Participants

**Fig. 1:**
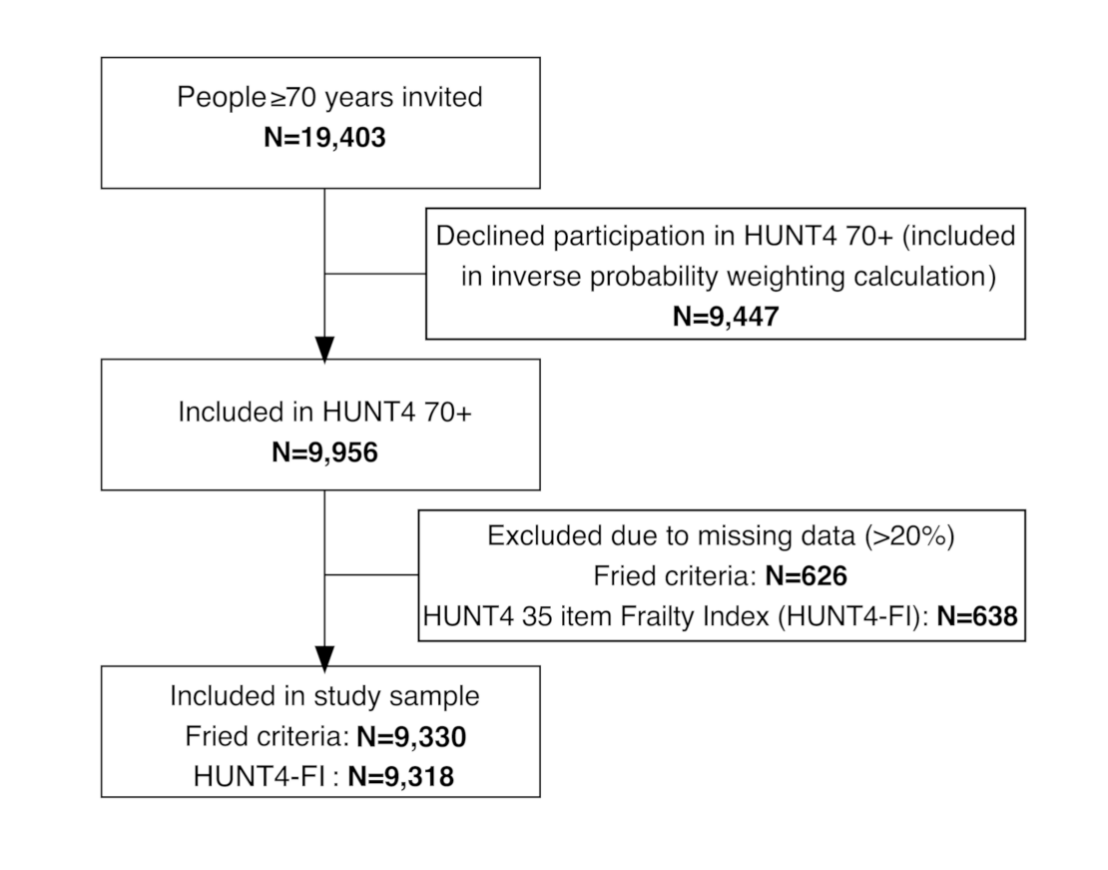
Analytical sample scheme

We used data from the fourth wave of the Trøndelag Health Study (HUNT), one of the largest population-based health studies worldwide, conducted in the former Nord-Trøndelag County, Central Norway [12]. This district consists of small towns and rural areas. In the fourth wave of HUNT, an additional examination of participants ≥ 70 years was conducted (HUNT4 70+). All 19,403 inhabitants ≥ 70 years living in Nord-Trøndelag County were invited by mail and eligible for inclusion in HUNT4 70+. In total, 9956 (51.3%) adults aged 70–103 years consented to participate and were included. The data were collected from September 2017 to March 2019. Flow-chart of the sample is shown in Fig. 1.

### Study design and data collection

This was a cross-sectional observational study. Participants completed self-report forms and underwent clinical examinations, face-to-face interviews and laboratory tests by healthcare professionals who had undergone a two-day training in the HUNT protocol. Field stations were established in all 23 municipalities. Additionally, participation was offered in private homes and nursing homes for those not able to attend the field station. Most participants (85.8%) were assessed at field stations, 7.8% in their own home, and 6.4% in nursing homes. All participants were asked to fill out two questionnaires which is the main source for self-reported data in both frailty models. However, given the high prevalence of cognitive impairment and dementia in Norwegian nursing homes, the HUNT4 70 + also used an adapted protocol in nursing homes that provided supplementary information from health personnel who knew the residents well. For consistency, we chose to use information regarding sleep, physical activity level, anxiety, depression, appetite and oral health from this adapted protocol for all nursing home residents, regardless of their cognitive status. Further details are described in Supplementary Tables 3 and 4.

For participants residing in nursing homes, written consent was requested to conduct a telephone interview with their next of kin. The same procedure applied to participants in the field stations/homes who reported subjective memory problems or who scored below age-related cut-off values on cognitive tests. We also used information from these interviews as sources of supplementary information about functional level, neuropsychiatric symptoms and cognitive difficulties.

### Procedures and assessments

For assessment of physical frailty, we used Fried criteria [4]. To assess frailty according to the deficit accumulation model, we constructed a 35-item FI, named HUNT4-FI. Both Fried criteria and FI are widely used and highly valued in research and clinical practice [13].

#### Fried criteria

The Fried criteria comprise five items: grip strength, gait speed, exhaustion, low physical activity and unintentional weight loss [4]. Grip strength was measured with a JAMAR Plus + digital dynamometer. The participant had three attempts on both hands, with the best result counting. Preferred gait speed was measured over 4 m with a static start. The participants were tested twice, gait speed (m/s) was calculated by using the fastest time from those two tests. Self-reported data on unintentional weight loss, physical activity and exhaustion were collected via face-to-face interviews, or via information from staff in nursing homes. Participants who met one or two of Fried criteria were categorised as pre-frail, and from three to five as frail in accordance with the original protocol [4]. Participants with fewer than four valid items were excluded from the statistical analysis (Fig. 1). In total, 9330 participants ( 93.7%) had sufficient information to be included in analyses based on Fried criteria. A detailed description of variables, cut-off values and compliance with Fried’s original protocol is available in Supplementary Table 3.

#### Construction of HUNT4-FI

The HUNT4-FI was constructed in accordance with the original procedure for creating a FI [5, 14] and recently updated recommendations [15]. We identified 35 items in the HUNT4 70 + dataset that met the criteria for constructing a FI. These included 11 laboratory markers, 14 clinical assessment items, and ten self-reported items. Supplementary Table 4 contains detailed information on construction, variables, cut-off values and scoring. Participants with > 20% missing HUNT4-FI values were excluded from the analyses. In total, 9318 participants (93.6%) had sufficient information to be included in the HUNT4-FI analyses (Fig. 1). For presentation purposes and best possible basis for comparison with the Fried criteria, the HUNT4-FI score was also converted to a categorical variable with the following cut-off values: Robust: <0.15, pre-frail: 0.15–0.24, frail: ≥0.25 in accordance with previous studies [16, 17].

### Demographic characteristics

Education is reported as elementary school ( ≤ 9 years), secondary school (10–12 years) and college/university (≥13years) retrieved from the National Education Database from Statistics Norway [18]. Information regarding cohabitation and municipal health services (defined as receiving home assistance, home nursing or being a nursing home resident) was based on self-report.

### Statistical analysis

Descriptive statistics for the total sample and for each group were calculated with means, standard deviations, frequencies and percentages. Differences between subgroups for continuous outcomes were analysed using t-tests, and chi-squared tests for categorical outcomes. To develop national estimates for prevalence of frailty in Norway for year 2019, we performed inverse probability weighting (IPW) in a two-step procedure, in line with a previous HUNT study [19]. First, we adjusted the prevalence estimates for non-responders in our sample; all eligible participants invited to HUNT4 70+, *N* = 19,403. For Fried criteria, we had 10073 non-responders, for HUNT4-FI the number was 10,085. This step allowed us to estimate representative prevalence of frailty and pre-frailty on a regional level (Nord-Trøndelag). Secondly, calibration using post-stratification weights and aggregated register data for Norway for year 2019 according to age (70–74, 75–79, 80–84, 85–89, 90–94, 95+), sex, and education (primary (≤ 9 years); secondary (10–12 years); tertiary (≥ 13 years) was performed and made it possible to present national estimates based on the regional data from Nord-Trøndelag.

Nord-Trøndelag lacks large cities, has a low immigration population and a lower educational level compared to Norway as total, while general health and life expectancy is on national average, and is considered to be representative of Norway [12, 20, 21]. Future projections of frailty due to population ageing in Norway in the coming decades were estimated by fixating the standardised prevalence of frailty in 2019 by age and sex. Finally, we multiplied our prevalence data with population projection data (main alternative) from Statistics Norway [22] by the same age groups and sex for the years 2023, 2030 and 2040. Analyses were conducted in STATA 18.

## Results

Table 1 presents demographic and clinical characteristics of the total sample, sorted by frailty status for participants included in Fried criteria sample and the HUNT4-FI sample. More women than men were classified as frail, regardless of frailty models (*p* < 0.001). Participants who were classified as frail were older, less educated, had lower scores on the Montreal Cognitive Assessment (MoCA) and slower gait speed than those classified as robust or pre-frail (all *p* < 0.001). Participants classified as frail, regardless of the frailty models, were also more likely to live alone, to receive municipal health services or being a nursing home resident compared to their robust or pre-frail counterparts (all *p* < 0.001). According to HUNT4-FI, frail participants had significantly higher body mass index (BMI) than robust or pre-frail participants (*p* < 0.001). According to Fried criteria, frail participants had significantly higher BMI than robust participants (*p* < 0.001), but not compared to pre-frail participants (*p* = 0.69).

**Table 1:** Characteristics of the fried criteria sample and the HUNT4-FI sample

	Fried criteria (N = 9330)			HUNT4-FI (N = 9318)		
	Robust N = 4609	Pre-frail N = 3789	Frail N = 932	Robust N = 3100	Pre-frail N = 3160	Frail N = 3058
Mean age	75.6 (4.5)	78.6 (6.4)	84.0 (7.5)	75.1 (4.1)	76.8 (5.1)	81.2 (7.3)
**Sex**						
Women	2228 (48.3)	2191 (57.8)	608 (65.2)	1534 (49.5)	1668 (52.8)	1819 (59.5)
Men	2381 (51.7)	1598 (42.2)	324 (34.8)	1566 (50.5)	1492 (47.2)	1239 (40.5)
**Age groups**						
70–74	2579 (56.0)	1470 (38.8)	154 (16.5)	1876 (60.5)	1498 (47.4)	831 (27.2)
75–79	1278 (27.7)	1006 (26.6)	154 (16.5)	839 (27.1)	932 (29.5)	667 (21.8)
80–84	576 (12.5)	683 (18.0)	192 (20.6)	305 (9.8)	494 (15.6)	640 (20.9)
85–89	153 (3.3)	417 (11.0)	230 (24.7)	71 (2.3)	192 (6.1)	528 (17.3)
90–94	21 (0.5)	168 (4.4)	159(17.1)	8 (0.3)	42 (1.3)	302 (9.9)
95+	1 (0.0)	45 (1.2)	43 (4.6)	1 (0.0)	2 (0.1)	90 (2.9)
**Education**						
≤ 9 years	797 (17.3)	1154 (30.5)	387 (41.5)	455 (14.7)	719 (22.8)	1172 (38.3)
10–12 years	2555 (55.5)	2017 (53.2)	455 (48.8)	1676 (54.0)	1810 (57.3)	1535 (50.2)
≥ 13 years	1256 (27.3)	618 (16.3)	90 (9.7)	969 (31.3)	631(19.9)	351 (11.5)
**Test location**						
Field station	4558 (98.9)	3337 (88.1)	416 (44.6)	3077 (99.3)	3087 (97.7)	2190 (71.6)
Home	23 (0.5)	278 (7.3)	271 (29.1)	8 (0.3)	60 (1.9)	468 (15.3)
Nursing home	9 (0.2)	168 (4.4)	244 (26.2)	0 (0.0)	5 (0.2)	397 (13.0)
Missing	19 (0.4)	6 (0.2)	1 (0.1)	15 (0.5)	8 (0.3)	3 (0.1)
**Living alone**						
Yes	1430 (31.0)	1587 (41.9)	518 (55.6)	915 (29.5)	1133 (35.9)	1413 (46.2)
Missing	26 (0.6)	142 (3.8)	99 (10.6)	16 (0.5)	35 (1.1)	294 (9.6)
**Receiving municipal health services**						
Yes	80 (1.7)	558 (14.7)	526 (56.4)	35 (1.1)	129 (4.1)	934 (30.5)
Missing	476 (10.3)	512(13.5)	124 (13.3)	286 (9.2)	394 (12.5)	491 (16.1)
**Clinical characteristics**						
Gait speed m/s	1.09 (0.22)	0.87 (0.26)	0.55 (0.2)	1.1 (0.23)	0.99 (0.25)	0.77 (0.27)
Body Mass Index	26.8 (3.8)	27.6 (4.6)	27.7 (5.9)	26.3 (3.4)	27.4 (4.3)	27.9 (5.2)
MoCA-score	23.9 (3.6)	22.1 (4.8)	17.9 (6.3)	24.9 (2.9)	23.3 (3.6)	19.5 (5.5)
HUNT4 FI-score	0.15 (0.07)	0.25 (0.1)	0.40 (0.11)	0.10 (0.03)	0.19 (0.03)	0.35 (0.09)

Continuous variables are expressed as mean (SD), categorical variables as N (%), and N for each variable is available in Supplementary Table 2. MoCA = Montreal Cognitive Assessment

The HUNT4-FI score ranged from zero to 0.76. The mean score was higher for women than men (0.22 (± 0.11) and 0.20 (± 0.11), respectively). Nursing home residents had a higher mean HUNT4-FI score than community-dwellers (0.45 (± 0.09) and 0.20 (± 0.10), respectively; *p* < 0.001).

Table 2 presents prevalence of frailty on a national level sorted by sex, age and frailty measurement. The prevalence of frailty in people ≥ 70 years in Norway in 2019 was 11.7% (95% confidence interval (CI) 11.0-12.4) according to Fried criteria and 36.0% (95% CI 35.1–36.9) according to HUNT4-FI. National prevalence of pre-frailty was 41.7% (95% CI 40.7–42.7) as measured by Fried criteria and 33.0% (95% CI 32.1–34.0) as measured by HUNT4-FI.

**Table 2:** Prevalence of frailty in Norway 2019* sorted by sex and age

	Fried criteria (N = 9330)			HUNT4-FI (N = 9318)		
	**Robust** **prevalence** % (95% CI)	**Pre-frail** prevalence % (95% CI)	Frail Prevalence % (95% CI)	Robust Prevalence % (95% CI)	Pre-frail Prevalence % (95% CI)	Frail Prevalence % (95% CI)
Total						
70–74	61.0 (59.5–62.4)	35.2 (33.8–36.7)	3.8 (3.2–4.4)	44.3 (42.8–45.8)	35.5 (34.1–37.0)	20.2 (19.0-21.4)
75–79	52.0 (50.0-53.9)	41.6 (39.7–43.6)	6.4 (5.5–7.4)	34.2 (32.3–36.1)	38.2 (36.3–40.2)	27.6 (25.9–29.4)
80–84	39.4 (36.9–41.9)	47.2 (44.6–49.8)	13.4 (11.7–15.3)	21.4 (19.4–23.6)	34.1 (31.6–36.6)	44.5 (42.0-47.1)
85–89	19.1 (16.5–22.0)	52.2 (48.8–55.7)	28.7 (25.6–31.9)	8.9 (7.1–11.0)	24.4 (21.6–27.6)	66.7 (63.4–69.9)
90–94	6.8 (4.4–10.2)	48.2 (42.8–53.7)	45.0 (39.7–50.4)	2.2 (1.1–4.3)	12.8 (9.6–16.9)	85.0 (80.8–88.4)
95+	2.2 (0.5–8.9)	50.9 (40.5–61.2)	46.9 (36.2–57.9)	0.9 (0.1–6.1)	1.8 (0.4–6.9)	97.3 (91.8–99.1)
Overall	46.6 (45.7–47.5)	41.7 (40.7–42.7)	11.7 (11.0-12.4)	31.0 (30.1–31.9)	33.0 (32.1–34.0)	36.0 (35.1–36.9)
**Women**						
70–74	57.5 (55.4–59.5)	38.0 (36.0-40.1)	4.5 (3.7–5.5)	42.5 (40.5–44.5)	36.1 (34.1–38.2)	21.5 (19.8–23.2)
75–79	47.7 (45.0-50.4)	44.6 (41.9–47.4)	7.7 (6.4–9.3)	32.2 (29.7–34.7)	37.6 (35.0-40.3)	30.2 (27.8–32.8)
80–84	33.8 (30.6–37.2)	50.6 (47.1–54.1)	15.6 (13.2–18.3)	18.3 (15.8–21.2)	33.8 (30.6–37.3)	47.8 (44.3–51.3)
85–89	16.3 (13.3–19.8)	53.3 (48.9–57.6)	30.4 (26.6–34.6)	7.2 (5.2–9.9)	23.3 (19.8–27.3)	69.5 (65.3–73.4)
90–94	5.0 (2.7–9.2)	48.9 (42.1–55.7)	46.1 (39.4–52.9)	1.8 (0.7–4.8)	9.4 (6.1–14.3)	88.8 (83.6–92.4)
95+	0	51.3 (39.1–63.4)	48.7 (36.6–60.9)	0	2.3 (0.6–8.8)	97.7 (91.2–99.4)
Overall	41.1 (39.8–42.3)	44.8 (43.4–46.2)	14.2 (13.2–15.2)	27.7 (26.6–28.8)	32.0 (30.8–33.3)	40.3 (39.1–41.5)
**Men**						
70–74	64.6 (62.5–66.6)	32.4 (30.4–34.4)	3.0 (2.4–3.9)	46.2 (44.1–48.3)	34.9 (32.8–37.0)	18.9 (17.3–20.7)
75–79	56.8 (54.0-59.6)	38.3 (35.5–40.7)	4.9 (3.8–6.4)	36.5 (33.7–39.3)	38.9 (36.1–41.8)	24.6 (22.2–27.2)
80–84	46.7 (42.9–50.5)	42.8 (39.1–46.6)	10.5 (8.4–13.1)	25.4 (22.2–28.9)	34.3 (30.8–38.1)	40.2 (36.6–44.0)
85–89	23.7 (19.2–28.8)	50.5 (44.8–56.2)	25.8 (21.1–31.1)	11.6 (8.5–15.7)	26.2 (21.6–31.6)	62.2 (56.6–67.5)
90–94	10.5 (5.7–18.3)	46.8 (37.8–56.0)	42.7 (34.4–51.5)	2.8 (1.1–7.2)	20.3 (14.0-28.4)	76.9 (68.5–83.5)
95+	9.9 (2.3–34.4)	49.3 (31.1–67.7)	40.8 (20.5–64.7)	4.2 (0.6–24.1)	0	95.8 (75.9–99.4)
Overall	53.3 (51.9–54.7)	38.0 (36.6–39.5)	8.6 (7.8–9.5)	35.0 (33.7–36.4)	34.3 (32.9–35.7)	30.7 (29.4–32.0)

*Standardisation done in a two-step process: (1) Weighted (inverse probability weighting) to account for non-response by sex, age, municipality, and nursing home. (2) Standardised (calibrated) to correspond to the distribution in Norway according to sex, age, and education in 2019

**Fig. 2:**
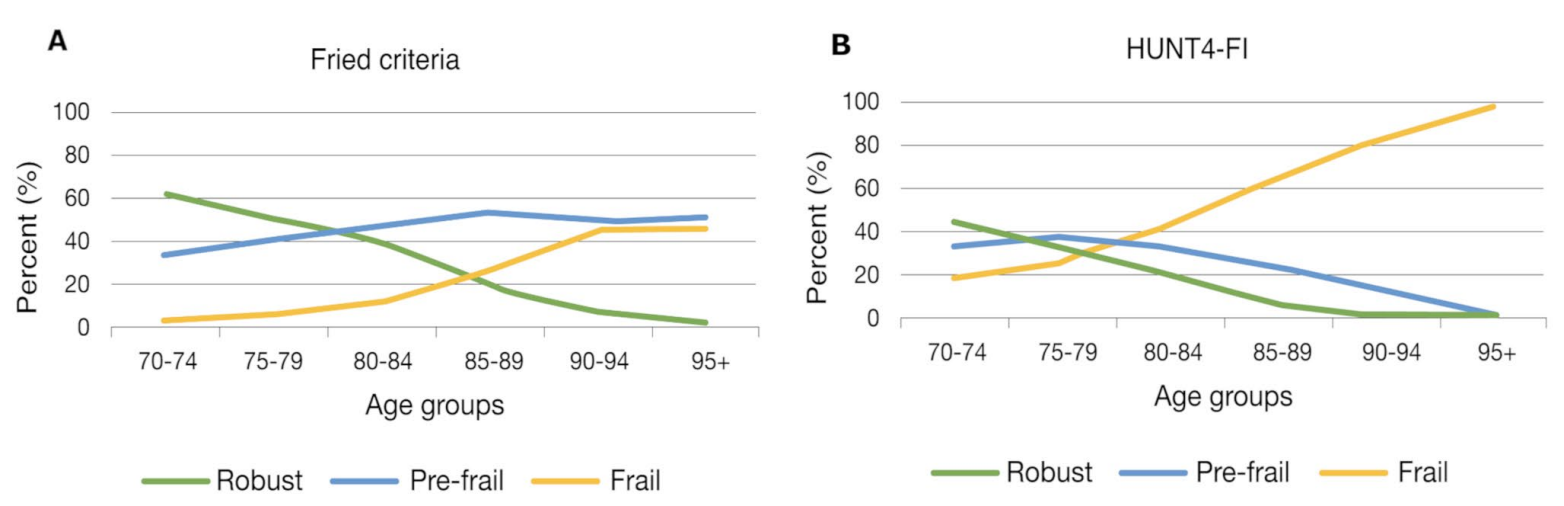
Prevalence of frailty by age. The margin plot depicts how the proportion of participants in each age group is classified as robust, pre-frail, or frail according to Fried criteria **(A)** and HUNT4-FI **(B)**

The prevalence of frailty increased with age (p-trend < 0.001), with a steeper curve from the age of 80–84 according to Fried criteria and from the age of 75–79 according to HUNT4-FI (Fig. 2). While there was a slight decrease in prevalence of pre-frailty from the age of 75–85 according to HUNT4-FI, prevalence of pre-frailty was slightly increasing until age of 85–89 according to Fried criteria.

**Fig. 3:**
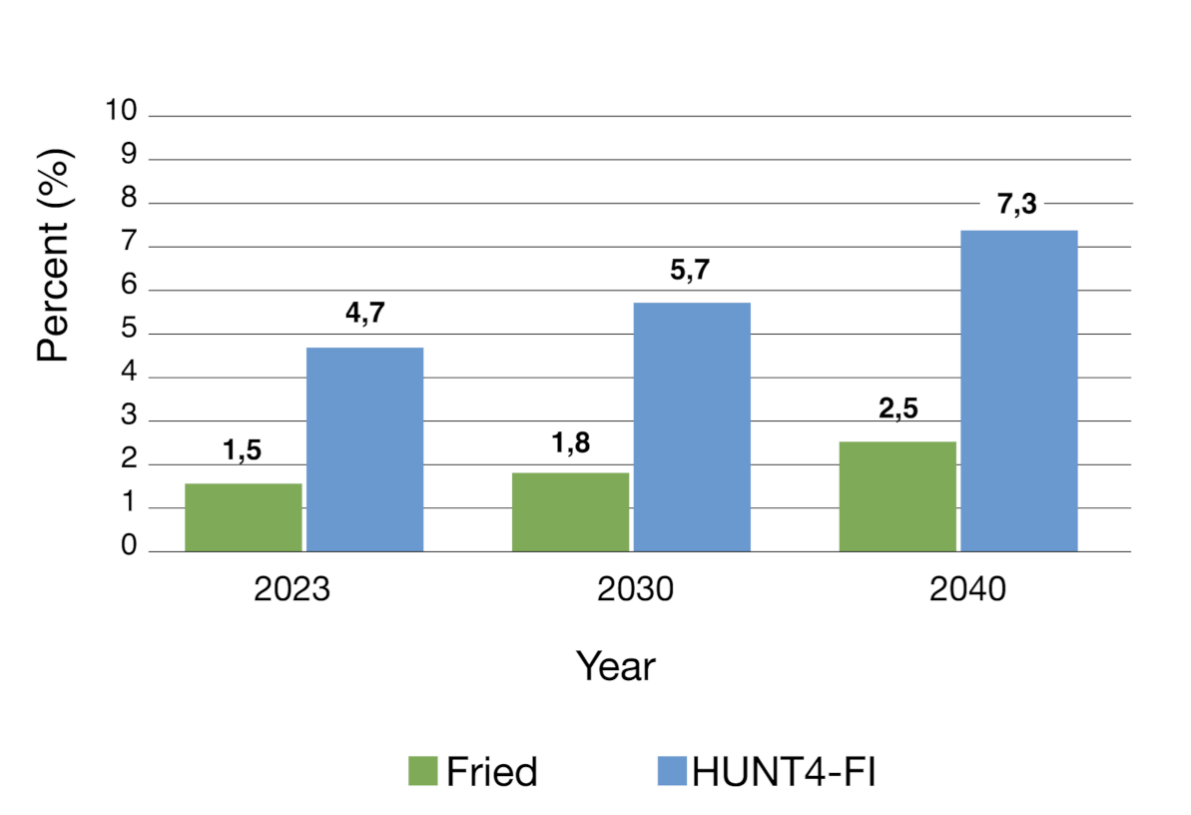
Current and future estimates, proportion of older people living with frailty out of the Norwegian population. Standardised prevalence of frailty in 2019 by age and sex, multiplied population projection data from Statistics Norway (main alternative) by the same age groups and sex for the years 2023, 2030 and 2040

Figure 3 shows estimates of the proportion of older people with frailty in the Norwegian population for 2023, 2030 and 2040. According to Fried criteria and HUNT4-FI, we estimate that older people with frailty accounted for 1.5% and 4.7%, respectively, of the total Norwegian population by 2023. This will increase to 2.5% (Fried criteria) and 7.3% (HUNT4-FI) by 2040.

## Discussion

In this large population-based study in Norway, 11.7% of adults ≥ 70 years were classified as frail, and 41.7% as pre-frail according to Fried criteria. Corresponding proportions using HUNT4-FI were 36.0% and 33.0%. Irrespective of frailty criteria used, prevalence was higher in women than in men, in nursing home residents than among community-dwellers and increased with age. According to demographic projections the proportion of older people living with frailty in the overall population in Norway will rise significantly during the next 17 years.

We found higher prevalence of frailty according to both models compared to previous Nordic [8–11] and European [7, 23] estimates. This indicates that health authorities should anticipate a greater proportion of older people at risk of functional decline and dependency than previously assumed. However, we found lower prevalence of frailty according to Fried criteria in the youngest age groups than in similar age groups reported from Europe overall, but in line with Sweden, Switzerland, Germany and Denmark [23]. This supports previous research showing a strong relationship between a country’s economic factors and its prevalence of frailty among middle-aged and older people [24], particularly in people < 80 years [25]. It is well-established that frailty is closely linked to multimorbidity [26], and a recent study found later onset of age-related diseases in Western European populations compared to the rest of Europe [27]. Taken together, our findings suggest that Norway and comparable countries in Western Europe should expect most people aged 70–79 to be robust and merely require efforts to help preserve and strengthen mental and physical reserves to prevent and postpone frailty. The overall higher prevalence of frailty in our study compared to previous studies is likely due to the efforts made in HUNT4 70 + to facilitate participation in the entire 70 + population. The way the data collection was designed and carried out, most likely resulted in a more representative sample in terms of age and function compared to previous European studies.

Those in our overall sample with inadequate data to be included in the final frailty analyses were older, less educated, a higher proportion received municipal health services, and there were more women than men (*p* < 0.001) (Supplementary Table 1). These are all factors associated with frailty [1]. Consequently, our findings may be interpreted as conservative estimates.

It is debatable whether frailty is understood as a precursor to functional impairment and need for assistance, or whether the condition itself includes functional limitations [1]. Participants considered frail according to Fried criteria had more functional limitations than did those considered frail according to HUNT4-FI (Table 1). Hence, the threshold for being categorised as frail seems to be higher using Fried criteria than when using HUNT4-FI. This finding is in line with previous studies [28, 29]. Additionally, HUNT4-FI appears to capture more men living with frailty than does Fried criteria. These findings support what prior studies have stated: the divergent operationalisations of frailty should be understood as complementary models with different strengths and limitations, and which to prefer depends on the purpose, population and setting [30, 31]. There is well-established evidence that frailty is associated with lower education [32], and our sample is no exception. Frail participants, regardless of criteria, had less education than robust or pre-frail ones. This highlights education`s impact on health diversity in old age even in high-income countries like Norway. Furthermore, it underscores the necessity for understanding and addressing modifiable risk factors earlier in life.

Due to the strong link between frailty and high healthcare costs [1], projection models for frailty should be given significant consideration. These data allow us to plan and assess the benefits of prevention and management efforts. However, future predictions of frailty prevalence should be regarded with caution because they are based on the premise that the age- and sex- specific prevalence (%) of frailty fixed at 2019 levels would remain constant in the future. Thus, there are uncertainties in our estimates. It is not accounted for if later born cohorts have lower levels of frailty than in this study. Previous population-based studies have reported that more recent born generations of older Norwegians perform better in terms of cognition [33] and grip strength [34]. Most likely, educational level will rise in more recent cohorts, and these factors could have a beneficial impact on our estimates. On the other side, the increasing prevalence of overweight, obesity and diabetes in Norway [12] may affect our estimates. All of these factors have been linked to higher levels of frailty [35, 36]. Considering these uncertainties, the results still stress the huge challenges posed by the ongoing demographic shift [1]. In 2040, we expect that 25 per 1000 Norwegians will be people ≥ 70 years living with frailty (Fried criteria), corresponding to 73 per 1000 according to HUNT4-FI. Our findings emphasise the significance of methodical planning that considers the great heterogeneity in health of the older part of the population. Addressing the age group 70–80 years in public health policies and research may be advisable to delay the sharp increase in frailty seen from the age of 80–84.

A strength of our study is the large population-based sample and the HUNT4 70 + design [12], which ensured inclusion of participants with a wide range in age and functioning, and our use of both dominant frailty models. Furthermore, our procedures for measuring frailty with Fried criteria are close to the original [4], and HUNT4-FI was created in accordance with updated recommended procedures [15].

There are several limitations in our study. Most HUNT participants were Caucasian, potentially limiting the generalisability of our findings to populations with greater ethnic diversity. Additionally, findings from Asia and America suggest that frailty prevalence is higher in rural areas [37]. The absence of large cities in Nord-Trøndelag may have influenced the frailty prevalence; however, there is limited evidence from European studies on this topic. Although our projections account for changes in age and sex distribution, they do not account for future shifts in health, lifestyle, and environmental factors within the population. There is no agreement on an operational definition of pre-frailty, and ongoing research aimed at determining the best measurement tools for identifying pre-frailty have high priority [38]. Our methods of using sub-threshold scores on Fried criteria and HUNT4-FI to classify pre-frailty may not be the most accurate tool to identify pre-frailty.

## Conclusion

We estimated frailty prevalence rates and future projections by analysing a large sample of representative data from 50% of all residents aged 70 and older in a geographical region of Norway. We found higher overall prevalence of frailty according to both dominant frailty models, compared to previous European estimates. We provide reliable estimates for governments to facilitate the planning of sustainable healthcare systems in the coming decades. Currently, our projections pose a substantial challenge to a society where health resources are already under strain. These findings accentuate the need for further research on modifiable risk factors in a life-course approach as a foundation for effective interventions to prevent and postpone frailty.

## Electronic supplementary material

Below is the link to the electronic supplementary material.

Supplementary Material 1

## Data Availability

Data for this study were provided by The Trøndelag Health Study (HUNT), available at https://www.ntnu.edu/hunt. Access to HUNT data analysis is open to research groups with a Principal Investigator affiliated with a Norwegian research institute. Non-Norwegian groups must collaborate with a partner in Norway for data use. Approval from the HUNT Data Access Committee (DAC), Regional Committee for Medical and Health Research Ethics, and sometimes the Data Inspectorate is required for each study. Participant data is not publicly accessible to maintain confidentiality.
