# Correction: Prevalence and future estimates of frailty and pre-frailty in a population-based sample of people 70 years and older in norway: the HUNT study

**DOI:** 10.1007/s40520-025-03165-7

**Published:** 2025-09-16

**Authors:** Ingebjørg Lavrantsdatter Kyrdalen, Heine Bjørn Strand, Geir Selbaek, Pernille Thingstad, Heidi Ormstad, Emiel O. Hoogendijk, Håvard Kjesbu Skjellegrind, Gro Gujord Tangen

**Affiliations:** 1 The Norwegian National Centre for Ageing and Health, Vestfold Hospital Trust, Tønsberg, Norway; 2 Institute of Clinical Medicine, Faculty of Medicine, University of Oslo, Oslo, Norway; 3 Norwegian Institute of Public Health, Oslo, Norway; 4 Department of Geriatric Medicine, Oslo University Hospital, Oslo, Norway; 5 Pernille Thingstad Trondheim Municipality, Trondheim, Norway; 6 Department of Public Health and Nursing, Faculty of Medicine and Health Sciences, HUNT Research Centre, Norwegian University of Science and Technology, Levanger, Norway; 7 University of South-Eastern Norway, Drammen, Norway; 8 Department of Epidemiology & Data Science, Department of General Practice, Amsterdam UMC – location VU University Medical Center, Amsterdam, The Netherlands; 9 Norway Levanger Hospital, Nord-Trøndelag Hospital Trust, Levanger, Norway; 10 Department of Rehabilitation Science and Health Technology, Oslo Metropolitan University, Oslo, Norway

**Aging Clinical and Experimental Research (2024) 36:188**

10.1007/s40520-024-02839-y

In the originally published version of this article, an error originating from the provided dataset affected the calculation of grip strength, leading to an overestimation of grip strength for approximately 2,800 participants. This discrepancy arose due to a merging error by our data provider, HUNT databank, when transferring the registration fields for kilograms and grams from the paper-based protocol to digital format. The two-digit precision for the gram value was not considered in this process. Consequently, this resulted in deviations ranging from 1 to 9 kg.

After re-running the analyses with the corrected data, the overall prevalence of frailty according to the Fried criteria increased from 10.6 to 11.7%, and according to HUNT4-FI, from 35.8 to 36.0%.

The main findings and conclusions of the article remain unchanged.

**Table 1:** Characteristics of the fried criteria sample and the HUNT4-FI sample

	Fried criteria (*N* = 9330)			HUNT4-FI (*N* = 9318)		
	Robust *N* = 4609	Pre-frail *N* = 3789	Frail *N* = 932	Robust *N* = 3100	Pre-frail *N* = 3160	Frail *N* = 3058
Mean age	75.6 (4.5)	78.6 (6.4)	84.0 (7.5)	75.1 (4.1)	76.8 (5.1)	81.2 (7.3)
**Sex**						
Women	2228 (48.3)	2191 (57.8)	608 (65.2)	1534 (49.5)	1668 (52.8)	1819 (59.5)
Men	2381 (51.7)	1598 (42.2)	324 (34.8)	1566 (50.5)	1492 (47.2)	1239 (40.5)
**Age groups**						
70–74	2579 (56.0)	1470 (38.8)	154 (16.5)	1876 (60.5)	1498 (47.4)	831 (27.2)
75–79	1278 (27.7)	1006 (26.6)	154 (16.5)	839 (27.1)	932 (29.5)	667 (21.8)
80–84	576 (12.5)	683 (18.0)	192 (20.6)	305 (9.8)	494 (15.6)	640 (20.9)
85–89	153 (3.3)	417 (11.0)	230 (24.7)	71 (2.3)	192 (6.1)	528 (17.3)
90–94	21 (0.5)	168 (4.4)	159(17.1)	8 (0.3)	42 (1.3)	302 (9.9)
95+	1 (0.0)	45 (1.2)	43 (4.6)	1 (0.0)	2 (0.1)	90 (2.9)
**Education**						
≤ 9 years	797 (17.3)	1154 (30.5)	387 (41.5)	455 (14.7)	719 (22.8)	1172 (38.3)
10–12 years	2555 (55.5)	2017 (53.2)	455 (48.8)	1676 (54.0)	1810 (57.3)	1535 (50.2)
≥ 13 years	1256 (27.3)	618 (16.3)	90 (9.7)	969 (31.3)	631(19.9)	351 (11.5)
**Test location**						
Field station	4558 (98.9)	3337 (88.1)	416 (44.6)	3077 (99.3)	3087 (97.7)	2190 (71.6)
Home	23 (0.5)	278 (7.3)	271 (29.1)	8 (0.3)	60 (1.9)	468 (15.3)
Nursing home	9 (0.2)	168 (4.4)	244 (26.2)	0 (0.0)	5 (0.2)	397 (13.0)
Missing	19 (0.4)	6 (0.2)	1 (0.1)	15 (0.5)	8 (0.3)	3 (0.1)
**Living alone**						
Yes	1430 (31.0)	1587 (41.9)	518 (55.6)	915 (29.5)	1133 (35.9)	1413 (46.2)
Missing	26 (0.6)	142 (3.8)	99 (10.6)	16 (0.5)	35 (1.1)	294 (9.6)
**Receiving municipal health services**						
Yes	80 (1.7)	558 (14.7)	526 (56.4)	35 (1.1)	129 (4.1)	934 (30.5)
Missing	476 (10.3)	512(13.5)	124 (13.3)	286 (9.2)	394 (12.5)	491 (16.1)
**Clinical characteristics**						
Gait speed m/s	1.09 (0.22)	0.87 (0.26)	0.55 (0.2)	1.1 (0.23)	0.99 (0.25)	0.77 (0.27)
Body Mass Index	26.8 (3.8)	27.6 (4.6)	27.7 (5.9)	26.3 (3.4)	27.4 (4.3)	27.9 (5.2)
MoCA-score	23.9 (3.6)	22.1 (4.8)	17.9 (6.3)	24.9 (2.9)	23.3 (3.6)	19.5 (5.5)
HUNT4 FI-score	0.15 (0.07)	0.25 (0.1)	0.40 (0.11)	0.10 (0.03)	0.19 (0.03)	0.35 (0.09)

Continuous variables are expressed as mean (SD), categorical variables as N (%), and N for each variable is available in Supplementary Table 2. MoCA = Montreal Cognitive Assessment

**Table 2:** Prevalence of frailty in Norway 2019* sorted by sex and age

	Fried criteria (*N* = 9330)			HUNT4-FI (*N* = 9318)		
	Robust prevalence % (95% CI)	Pre-frail prevalence % (95% CI)	Frail Prevalence % (95% CI)	Robust Prevalence % (95% CI)	Pre-frail Prevalence % (95% CI)	Frail Prevalence % (95% CI)
**Total**						
70–74	61.0 (59.5–62.4)	35.2 (33.8–36.7)	3.8 (3.2–4.4)	44.3 (42.8–45.8)	35.5 (34.1–37.0)	20.2 (19.0-21.4)
75–79	52.0 (50.0-53.9)	41.6 (39.7–43.6)	6.4 (5.5–7.4)	34.2 (32.3–36.1)	38.2 (36.3–40.2)	27.6 (25.9–29.4)
80–84	39.4 (36.9–41.9)	47.2 (44.6–49.8)	13.4 (11.7–15.3)	21.4 (19.4–23.6)	34.1 (31.6–36.6)	44.5 (42.0-47.1)
85–89	19.1 (16.5–22.0)	52.2 (48.8–55.7)	28.7 (25.6–31.9)	8.9 (7.1–11.0)	24.4 (21.6–27.6)	66.7 (63.4–69.9)
90–94	6.8 (4.4–10.2)	48.2 (42.8–53.7)	45.0 (39.7–50.4)	2.2 (1.1–4.3)	12.8 (9.6–16.9)	85.0 (80.8–88.4)
95+	2.2 (0.5–8.9)	50.9 (40.5–61.2)	46.9 (36.2–57.9)	0.9 (0.1–6.1)	1.8 (0.4–6.9)	97.3 (91.8–99.1)
Overall	46.6 (45.7–47.5)	41.7 (40.7–42.7)	11.7 (11.0-12.4)	31.0 (30.1–31.9)	33.0 (32.1–34.0)	36.0 (35.1–36.9)
**Women**						
70–74	57.5 (55.4–59.5)	38.0 (36.0-40.1)	4.5 (3.7–5.5)	42.5 (40.5–44.5)	36.1 (34.1–38.2)	21.5 (19.8–23.2)
75–79	47.7 (45.0-50.4)	44.6 (41.9–47.4)	7.7 (6.4–9.3)	32.2 (29.7–34.7)	37.6 (35.0-40.3)	30.2 (27.8–32.8)
80–84	33.8 (30.6–37.2)	50.6 (47.1–54.1)	15.6 (13.2–18.3)	18.3 (15.8–21.2)	33.8 (30.6–37.3)	47.8 (44.3–51.3)
85–89	16.3 (13.3–19.8)	53.3 (48.9–57.6)	30.4 (26.6–34.6)	7.2 (5.2–9.9)	23.3 (19.8–27.3)	69.5 (65.3–73.4)
90–94	5.0 (2.7–9.2)	48.9 (42.1–55.7)	46.1 (39.4–52.9)	1.8 (0.7–4.8)	9.4 (6.1–14.3)	88.8 (83.6–92.4)
95+	0	51.3 (39.1–63.4)	48.7 (36.6–60.9)	0	2.3 (0.6–8.8)	97.7 (91.2–99.4)
Overall	41.1 (39.8–42.3)	44.8 (43.4–46.2)	14.2 (13.2–15.2)	27.7 (26.6–28.8)	32.0 (30.8–33.3)	40.3 (39.1–41.5)
**Men**						
70–74	64.6 (62.5–66.6)	32.4 (30.4–34.4)	3.0 (2.4–3.9)	46.2 (44.1–48.3)	34.9 (32.8–37.0)	18.9 (17.3–20.7)
75–79	56.8 (54.0-59.6)	38.3 (35.5–40.7)	4.9 (3.8–6.4)	36.5 (33.7–39.3)	38.9 (36.1–41.8)	24.6 (22.2–27.2)
80–84	46.7 (42.9–50.5)	42.8 (39.1–46.6)	10.5 (8.4–13.1)	25.4 (22.2–28.9)	34.3 (30.8–38.1)	40.2 (36.6–44.0)
85–89	23.7 (19.2–28.8)	50.5 (44.8–56.2)	25.8 (21.1–31.1)	11.6 (8.5–15.7)	26.2 (21.6–31.6)	62.2 (56.6–67.5)
90–94	10.5 (5.7–18.3)	46.8 (37.8–56.0)	42.7 (34.4–51.5)	2.8 (1.1–7.2)	20.3 (14.0-28.4)	76.9 (68.5–83.5)
95+	9.9 (2.3–34.4)	49.3 (31.1–67.7)	40.8 (20.5–64.7)	4.2 (0.6–24.1)	0	95.8 (75.9–99.4)
Overall	53.3 (51.9–54.7)	38.0 (36.6–39.5)	8.6 (7.8–9.5)	35.0 (33.7–36.4)	34.3 (32.9–35.7)	30.7 (29.4–32.0)

*Standardisation done in a two-step process: (1) Weighted (inverse probability weighting) to account for non-response by sex, age, municipality, and nursing home. (2) Standardised (calibrated) to correspond to the distribution in Norway according to sex, age, and education in 2019

The orginal article has been updated.

## Supplementary Information

Below is the link to the electronic supplementary material.

Supplementary Material 1

