# Supplementary material for: Prevalence and future estimates of frailty and pre-frailty in a population-based sample of people 70 years and older in Norway: the HUNT study
Source: Aging Clin Exp Res. 2024 Sep 10;36(1):188. doi: 10.1007/s40520-024-02839-y (PMC11387435; doi:10.1007/s40520-024-02839-y)
Supplement: Supplementary file 1 — Supplementary Material 1 [file 40520_2024_2839_MOESM1_ESM.pdf]

Article Title: Prevalence and future estimates of frailty and pre-frailty in a population-based sample of persons 70 years and older in Norway: The HUNT Study

Authors: Ingebjørg Lavrantsdatter Kyrdalen, Msc, Bjørn Heine Strand, PhD, Geir Selbæk, PhD, Pernille Thingstad, PhD, Heidi Ormstad, PhD, Emiel O. Hoogendijk, PhD, Håvard Kjesbu Skjellegrind, PhD & Gro Gujord Tangen, PhD

Corresponding author: Ingebjørg L. Kyrdalen[1,2]

[1] The Norwegian National Centre for Ageing and Health, Tønsberg, Norway

[2] Faculty of Medicine, University of Oslo, Oslo, Norway

Supplementary Information

Table of Contents

Supplementary Table 1 Descriptive characteristics of participants in total sample (N=9956) included in frailty analyses versus participants excluded from analyses 2

Supplementary Table 2 Number of participants (N) for each variable included in descriptive analyses (Table 1) 2

Supplementary Table 3 Fried criteria variables used in this study 3

Supplementary method - construction of HUNT4-FI 4

Supplementary Table 4 HUNT4-FI variables with coding 4

Supplementary Table 5 Frailty projections for Norway 2023-2040 9

Supplementary Table 6 Correspondence in frailty categories between HUNT4-FI and Fried criteria in our sample 10

Supplementary Fig. 1 Distribution of HUNT4-FI sum score in the total sample (N=9318) 11

Supplementary Fig. 2 HUNT4-FI sum score sorted by sex (N=9318) 11

Stata-syntax HUNT4-FI 12

Stata syntax Fried criteria 21

References for cut-off values 23

Supplementary Table 1 Descriptive characteristics of participants in total sample (N=9956) included in frailty analyses versus participants excluded from analyses

	Fried Criteria analyses		HUNT4-FI analyses	
	Included N=9330	Excluded N=626	Included N=9318	Excluded N=638
Age	77.6 (6.2)	84.1 (7.7)	77.7 (6.2)	84.1 (7.5)
Women	5027 (53.9)	393 (62.5)	5021 (53.9)	399 (62.6)
Men	4303 (46.1)	233 (37.5)	4297 (46.1)	239 (37.5)
**Education**				
≤9 years	2338 (25.1)	280 (44.7)	2346 (25.2)	272 (42.7)
10–12 years	5027 (53.9)	289 (46.2)	5021 (53.9)	295 (46.3)
≥13 years	1964 (21.1)	57 (9.1)	1951 (20.9)	70 (11.0)
**Test location**				
Field station	8311 (89.3)	210 (34.0)	8354 (89.9)	167 (26.2)
Home	572 (6.2)	204 (32.6)	536 (5.8)	240 (37.6)
Nursing home	421 (4.5)	212 (33.9)	402 (4.3)	231 (36.2)
**Living alone**				
Yes	3535 (37.9)	131 (20.9)	3461 (37.1)	205 (32.1)
Missing	267 (2.9)	354 (56.6)	345 (3.7)	276 (43.3)
**Receiving municipal health services**				
Yes	1164 (12.5)	263 (42.0)	1098 (11.8)	329 (51.6)
Missing	1112 (11.9)	245 (39.1)	1171 (12.6)	186 (29.2)
**Clinical characteristics**				
Gait speed m/s	0.96 (0.25)	0.56 (0.28)	0.96 (0.28)	0.63 (0.27)
Body Mass Index	27.2 (4.4)	26.9 (5.1)	27.2 (4.4)	27.3 (5.1)
MoCA-score	22.7 (4.7)	15.9 (6.9)	22.7 (4.6)	15.5 (7.5)

Continuous variables are expressed as mean (SD), categorical variables as N (%).
MoCA= Montreal Cognitive Assessment. P-values for within-group differences for Body Mass Index: Fried Criteria (p=0.253*)* and HUNT4-FI (p=0.239*)*. For all other variables p=<0.001 regardless of frailty measurement.

Supplementary Table 2 Number of participants (N) for each variable included in descriptive analyses (Table 1)

	**Total sample (N=9956)**	**Fried criteria sample (N=9330)**	**HUNT4-FI criteria sample (N=9318)**
Age	9956	9324	9318
Sex	9956	9324	9318
Education	9956	9324	9318
Test location	9930	9298	9292
Living alone	9336	9058	8973
Receiving municipal health services	8600	8213	8147
Gait speed m/s	9143	8918	8805
Body Mass Index	9458	9065	9042
MoCA-score	9382	8973	9005

MoCA= Montreal Cognitive Assessment.

Supplementary Table 3 Fried criteria variables used in this study

	**Original Fried protocol [1]**	**Fried criteria with some modifications used in the present study**	**Missing (N) out of total sample (N=9956)**
Gait speed	Test method:<br>Time to walk (seconds) 15 feet at usual pace stratified by sex and height (gender-specific cut-off values at medium height):<br>Score 1 in Fried Criteria if:<br>Men ≤173 cm and women ≤159 cm: ≤0.6531 m/s<br>Men >173 cm and women >159 cm: ≤0.762 m/s	Test method:<br>Gait speed test from Short Physical Performance Battery (SPPB): walk 4 meters at usual pace. Fastest of two tests. Result converted from meters to feet. cut-off values as in the original Fried protocol [1]:<br>Men ≤173 cm and women ≤159 cm: ≤0.6531 m/s<br>Men >173 cm and women >159 cm: ≤0.762 m/s	328
Grip strength	Test method:<br>Measured by JAMAR dynamometer (kg). Maximal strength in dominant hands, three tests. Stratified by sex and BMI quartiles. Lowest 20 % were identified, giving the following cut-off values for frailty:<br>Men:<br><29 kg for BMI ≤24<br><30 kg for BMI 24.1–26<br><30 kg for BMI 26.1–28<br><32 kg for BMI >28<br>Women:<br><17 kg for BMI ≤23<br><17.3 kg for BMI 23.1–26<br><18 kg for BMI 26.1–29<br><21 kg for BMI >29	Test method:<br>Measured by JAMAR Plus + digital dynamometer. Maximal strength. three attempts each hand, best result counting.<br>Cut-off values as in the original Fried protocol [1]:<br>Men:<br><29 kg for BMI ≤24<br><30 kg for BMI 24.1–26<br><30 kg for BMI 26.1–28<br><32 kg for BMI >28<br>Women:<br><17 kg for BMI ≤23<br><17.3 kg for BMI 23.1–26<br><18 kg for BMI 26.1–29<br><21 kg for BMI >29	471
Weight loss	Participants are given score 1 if answering “yes” to the question*:*<br>“In the last year, have you lost more than 10 pounds (4.5 kg) unintentionally?”	Participants are given score 1 if answering “yes” to the question:<br>“The past six months, have you involuntarily lost more than 5 kg body weight?”<br>Or, if item is missing:<br>Body Mass Index <22 [2]	290
Level of physical activity	Minnesota Leisure Time Activity Questionnaire.<br>Kcal/week calculated, lowest 20% identified, giving the following cut-off values for frailty:<br>Men:<383 kcal of physical activity/week<br>Women: <270 kcal of physical activity/week	Community-dwellers are given score 1 if answering «Less than once a week" or "Never" to the question: "How often do you exercise?"<br>Nursing home residents are given score 1 if carer answering, "Can move with assistance" or "Cannot walk or move without assistance" or "Lying in bed more than half of the time" on P-ADL item Physical Movement	598
Exhaustion/tiredness	Items from the Center of Epidemiologic Studies Depression Scale:<br>1) I felt that everything I did was an effort.<br>2) I could not get going.<br>Question: “How often in the last week did you feel this way?”<br>Participants were given score 1 if answering “A moderate amount of time (3-4 days)” or “most of the time”	Participants are given score 1 if answering "yes" to the question "Do you feel exhausted/tired?"<br>If item is missing; answering "Somewhat tired and worn out" or "Tired and worn out" to the question «Do you feel. for the most part, strong and fit or tired and worn out?	556

Score 0=robust, 1-2=prefrail, 3-5=frail [1]

Supplementary method - construction of HUNT4-FI

FI is based on a comprehensive geriatric assessment that maps several deficits or conditions across organ systems that accumulate with ageing and are related to health and function [3]. HUNT4-FI was constructed in accordance with updated recommendations for creating a FI from an existing dataset [4].

Each item was given a score between 0 and 1 with 0 representing that the deficit was absent. If a deficit was present, most items were scored 1. For some ordinal or interval data we used the scores 0, 0.5 and 1.0 for three response levels, and 0, 0.25, 0.5, 0.75 and 1.0 for five response levels, in accordance with previous studies [4, 5].

Supplementary Table 4 HUNT4-FI variables with coding

	**HUNT4-FI code=0**	**HUNT4-FI code=1**	**Non-binary HUNT4-FI coding**	**Comments**	**Missing (N) out of total sample (N=9956)**
Albumin*	34–45	<34 or >45			586
Hemoglobin A1c (HbA1c) *	20–42	<20 or >42			488
Estimated Glomelural Filtration Rate (GFR) ml/min*	≥60	<60			417
C-Reactive Protein (CRP)*	<5	≥5			449
Hemoglobin*	Women: 11.7–15.3<br>Men: 13.4–17.0	Women: <11.7 or >15.3<br>Men: <13.4 or >17.0			483
Creatinin*	Women: 45–90<br>Men: 60–105	Women: <45 or >90<br>Men: <60 or >105			417
Thyroid-Stimulating Hormone(TSH)*	0.5–3.6	<0.5 or >3.6			423
Leukocytes*	4.1–9.8	≤4.1 or >9.8			483
Serum Cholesterol*	3.9–7.8	<3.9 or >7.8			417
Granulocytes distribution %*	1.8–6.9	<1.8 or >6.9			517
Triglycerides*	0.45–2.60	<0.45 or >2.60			429
Grip strength			Women: ≥20 kg=0, 16-19 kg=0.5, <16 kg=1<br>Men: ≥32 kg =0, 26-31 kg=0.5, <26 kg=1	Best result of three attempts counting.<br>Measured with JAMAR + digital dynamometer.<br>Cut-off values from Kim et.al [6]	570

	HUNT4-FI code=0	HUNT4-FI code=1	Non-binary HUNT4-FI coding	Comments	Missing (N) out of total sample (N=9956)
Gait speed 4-m usual pace			4 points=0, 3 points=0.25, 2 points=0.5, 1 point=0.75, 0 point or not able to walk=1	Best result of two attempts counting<br>Cut-off values as in SPPB protocol [7]	557
Five- times chair rise			4 points=0, 3 points=0.25, 2 points=0.5, 1 point=0.75, 0 point or not able to rise from a chair=1	Cut-off values as in SPPB protocol [7]	320
MoCA Language			5 points=0, 4–3 points=0.5, 2–0 points or performed SIB instead of MoCA=1		380
MoCA Attention			6–5 points=0, 4–2 points=0.5, 1–0 points or performed SIB instead of MoCA=1		326
MoCA Memory			6 points=0, 5–4 points=0.5, 3–0 points or performed SIB instead of MoCA=1		392
MoCA Orientation			6 points=0, 5–4 points=0.5, 3–0 points or performed SIB instead of MoCA=1		274
MoCA Visuospatial/executive function			5–4 points=0, 3 points=0.5, 2–0 points or performed SIB instead of MoCA=1		365
Systolic blood pressure	≤140	>140		Using mean of 2. and 3. measurement	164
Diastolic blood pressure	≤90	>90		Using mean of 2. and 3. measurement	164
Body Mass Index			22-29.9 =0, ≥30=0.5, <22=1	Cut-off value underweight [2]<br>Cut-off value obesity [8]	535
Pulse	60–99	<60 or >99		Using mean of 2. and 3. measurement	288
O²-saturation	≥96 [9]	<96 [9]		Using mean of 2. and 3. measurement	399
Dementia diagnosed by a medical doctor	No dementia	Dementia		Participants with insufficient information to make a diagnose scored as "missing".	206
How is your health at the moment?	Answers "good" or "excellent".	Answers "poor" or "not so good".		Self-reported in all participants, regardless of residence.	868

	HUNT4-FI code=0	HUNT4-FI code=1	Non-binary HUNT4-FI coding	Comments	Missing (N) out of total sample (N=9956)
Depressive symptoms	Answers "no" to questions about depressive symptoms**, or if primary variables are missing: total score HADS depression ≤8 [10].	Answers "yes" to questions about depressive symptoms**, or if primary variables are missing: total score HADS depression >8 [10].		Self-reported in community-dwelling participants, carer-reported in nursing home residents.	512
Symptoms of anxiety	Answers "no" to questions about symptoms of anxiety**, or if primary variables are missing: total score HADS anxiety ≤8 [10].	Answers "yes" to questions about symptoms of anxiety**, or if primary variables are missing; total score HADS anxiety >8 [10].		Self-reported in community-dwelling participants, carer-reported in nursing home residents.	558
Pain in mouth or teeth	Answers "No" to the question "Have you had pain or discomfort in your teeth or mouth during the last four weeks?"	Answers "Yes" to the question "Have you had pain or discomfort in your teeth or mouth during the last four weeks?"		Self-reported in community-dwelling participants, carer-reported in nursing home residents.	252
Exhaustion/tiredness current	Answers "no" to the question "Do you feel exhausted/tired?" or if this item is missing; answers "Very strong and fit", "Strong and fit", "Somewhat strong and fit" or "Somewhere in between" to the question «Do you feel, for the most part, strong and fit or tired and worn out?	Answers "yes" to the question "Do you feel exhausted/tired?" or if this item is missing; answers "Somewhat tired and worn out" or "Tired and worn out" to the question «Do you feel, for the most part, strong and fit or tired and worn out?		Self-reported in all participants, regardless of residence.	556
Bodily pain	Data required at least one of the two questions: Answers "No pain", "very mild" or "mild» to the question "How strong physical pain have you had during the last four weeks?" or "No" to the question "During the past 12 months, have you had pain in your joint that has	Data required at least one of the two questions: Answers "Moderate»", "Strong" or "Very strong" to the question "How strong physical pain have you had during the last four weeks?" or "Yes" to the question "During the past 12 months, have you had pain in your joint that has lasted for		Self-reported in all participants, regardless of residence.	695

	HUNT4-FI code=0	HUNT4-FI code=1	Non-binary HUNT4-FI coding	Comments	Missing (N) out of total sample (N=9956)
	lasted for more than six consecutive weeks?"	more than six consecutive weeks?"			
Appetite	Answers "No" to the question: "Have you had poor appetite during the last four weeks?"	Answers "Yes" to the question: "Have you had poor appetite during the last four weeks?"		Self-reported in community-dwelling participants, carer-reported in nursing home residents.	162
Chewing function	Answer "Yes" to the question "Can you chew all kinds of food?"	Answer "No" to the question "Can you chew all kinds of food?"		Self-reported in community-dwelling participants, carer-reported in nursing home residents.	214
Physical activity level	Community-dwellers: Answer "Once a week", "2-3 times a week", or "Nearly every day" to the question "How often do you exercise?" Nursing home residents: Answer "walks outdoor, in smooth and rough terrain" or "walks in the local environment" on item P-ADL Physical Movement.	Community-dwellers: Answer "Less than once a week" or "Never" to the question "How often do you exercise?" Nursing home residents: Answer "Can move with assistance" or "Cannot walk or move without assistance" or "Lying in bed more than half of the time" on item P-ADL Physical Movement.		Self-reported in community-dwelling participants, carer-reported in nursing home residents.	640

	HUNT4-FI code=0	HUNT4-FI code=1	Non-binary HUNT4-FI coding	Comments	Missing (N) out of total sample (N=9956)
Insomnia	Answers "never/seldom or "sometimes" to all three questions about sleep problems; difficulty falling asleep, repeatedly wakening up during the night, wake up too early. For nursing home residents: staff answers "No" to the question about presence of sleep and night-time behaviour on NPI-NH.	Answers "At least three times a week" to one or several of the three about sleep problems; difficulty falling asleep, repeatedly wakening up during the night, wake up too early. For nursing home residents: staff answers "Yes" to the question about presence of sleep and night-time behaviour on NPI-NH.		Self-reported in community-dwelling participants, carer-reported in nursing home residents.	712

Cut-off values for presentation in Prevalence paper: Robust: <0.15, prefrail: 0.15–0.24, frail: ≥0.25 [11-13]. *Reference values for laboratory markers in accordance with Nord-Trøndelag Hospital Trust [14]. ** CONOR Mental Health Index applied among community-dwellers, Neuropsychiatric Inventory Nursing Homes (NPI-NH) in nursing homes. MoCA= Montreal Cognitive Assessment, SIB= Severe Impairment Battery, SPPB= Short Physical Performance Battery, HADS= Hospital Anxiety and Depression Scale, NPI-NH= Neuropsychiatric Inventory Nursing Homes.

Supplementary Table 5 Frailty projections for Norway 2023-2040

Age	Total 2023	Men 2023	Women 2023	Total 2030	Men 2030	Women 2030	Total 2040	Men 2040	Women 2040
**Number of persons living with frailty according to Fried criteria, based on age and gender distribution for Norway***									
**Age**	**Total 2023**	**Men 2023**	**Women 2023**	**Total 2030**	**Men 2030**	**Women 2030**	**Total 2040**	**Men 2040**	**Women 2040**
70-74	9591	3780	5811	10474	4135	6339	12380	4959	7421
75-79	14062	5191	8870	14955	5591	9363	17408	6663	10745
80-84	17080	6020	11059	26049	9665	16384	29613	11192	18421
85-89	20096	7500	12596	29133	11735	17398	42510	17717	24793
90+	21294	6235	15059	24267	7963	16304	47092	17506	29587
Total	82123	28727	53396	104878	39091	65788	149004	58037	90967
**Number of persons living with frailty according to HUNT4-FI, based on age and gender distribution for Norway***									
**Age**	**Total 2023**	**Men 2023**	**Women 2023**	**Total 2030**	**Men 2030**	**Women 2030**	**Total 2040**	**Men 2040**	**Women 2040**
70-74	51579	23814	27765	56338	26052	30286	66699	31244	35456
75-79	60853	26062	34790	64794	28071	36723	75594	33451	42143
80-84	56937	23050	33887	87207	37005	50202	99293	42849	56444
85-89	48583	18081	30502	70423	28292	42131	102752	42713	60039
90+	41153	11809	29344	46851	15081	31770	90807	33153	57653
Total	259105	102816	156288	325613	134501	191112	435145	183411	251734
**Number of persons living with pre-frailty according to Fried Criteria, based on age and gender distribution for Norway***									
**Age**	**Total 2023**	**Men 2023**	**Women 2023**	**Total 2030**	**Men 2030**	**Women 2030**	**Total 2040**	**Men 2040**	**Women 2040**
70-74	89898	40824	49073	98189	44661	53528	116226	53560	62666
75-79	91956	40577	51379	97937	43704	54234	114317	52080	62237
80-84	60413	24541	35872	92541	39398	53143	105371	45621	59751
85-89	38072	14680	23392	55281	22970	32310	80723	34679	46044
90+	22918	6956	15962	26165	8883	17281	50889	19529	31361
Total	303256	127578	175678	370113	159617	210496	467527	205469	262058
**Number of persons living with pre-frailty according to HUNT4-FI, based on age and gender distribution for Norway***									
**Age**	**Total 2023**	**Men 2023**	**Women 2023**	**Total 2030**	**Men 2030**	**Women 2030**	**Total 2040**	**Men 2040**	**Women 2040**
70-74	90594	43974	46620	98959	48107	50852	117226	57693	59533
75-79	84528	41213	43315	90110	44388	45722	105365	52896	52469
80-84	43629	19667	23962	67072	31574	35498	76473	36560	39912
85-89	17929	7703	10226	26178	12054	14124	38326	18198	20128
90+	4892	2441	2451	5771	3118	2653	11669	6854	4815
Total	241572	114998	126573	288090	139241	148849	349058	172201	176857

*Men and women are added together to determine the total figures. The fact that the sex distribution in Norway changes throughout time is accounted for.

Supplementary Table 6 Correspondence in frailty categories between HUNT4-FI and Fried criteria in our sample

Fried criteria categories	HUNT4-FI categories			
	Robust	Pre-frail	Frail	Total
Robust	2554	1619	387	4560
Pre-frail	520	1436	1687	3643
Frail	4	48	744	796
Total	3085	3103	2818	8999

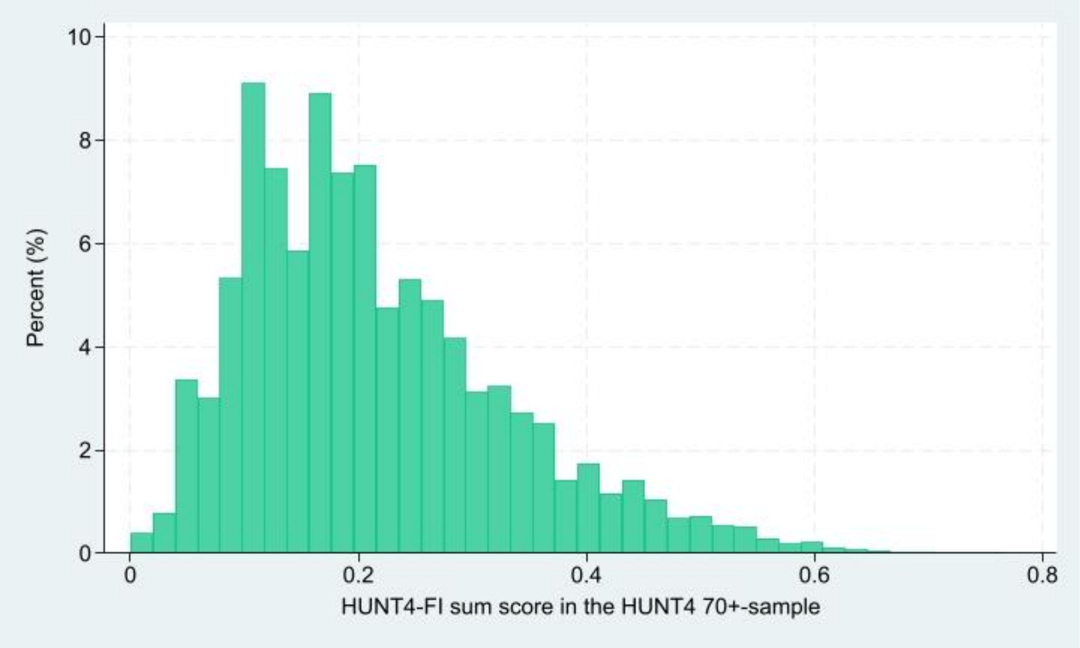

Supplementary Fig. 1 Distribution of HUNT4-FI sum score in the total sample (N=9318)

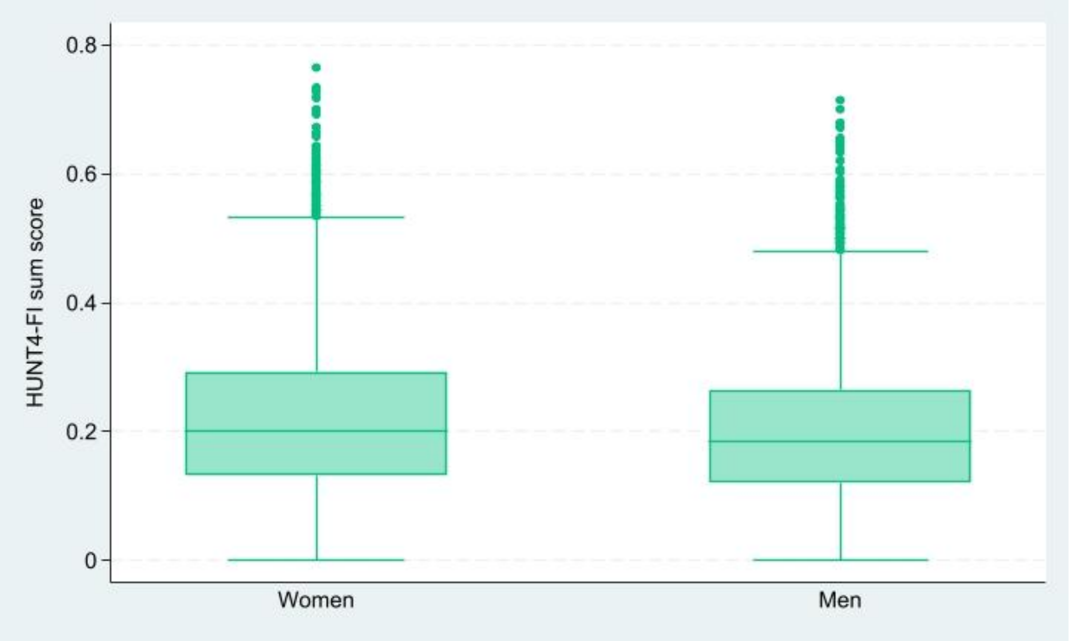

Supplementary Fig. 2 HUNT4-FI sum score sorted by sex (N=9318)

Stata-syntax HUNT4-FI

```
*------------------LAB-FI, ITEM 1-11-----------------------------------------------------*

*1) ALBUMIN*
*Values that were originally expressed in micromol, must be converted to grams per liter. Procedure in accordance with the analyzers producers: Gram/liter=micromol x 0.666*
generate Albumin_gr_liter= SeAlb_NT4BLM*0.066
summ Albumin_gr_liter

generate Albumin_FI=1 if Albumin_gr_liter <34
        replace Albumin_FI=1 if Albumin_gr_liter >=45 & Albumin_gr_liter<.
        replace Albumin_FI=0 if inrange(Albumin_gr_liter,34,45)
                tab Albumin_FI,missing


*2) HEMOGLOBINEA1c (HbA1c)*
gen HbA1c_FI=.
                replace HbA1c_FI=1 if BloHbA1cIFCC_NT4BLM <20
                replace HbA1c_FI=1 if BloHbA1cIFCC_NT4BLM>42 & BloHbA1cIFCC_NT4BLM<.
                replace HbA1c_FI=0 if inrange(BloHbA1cIFCC_NT4BLM,20,42)
tab HbA1c_FI, missing


*3) ESTIMATED GLOMELURAL FILTRATION RATE (GFR) ML/MIN*
gen GFR_FI=.
                replace GFR_FI=1 if GFREstCKD_NT4BLM <60
                replace GFR_FI=0 if GFREstCKD_NT4BLM >=60 & GFREstCKD_NT4BLM <.
tab GFR_FI, missing


*4) C-REACTIVE PROTEIN (CRP)*
gen CRP_FI=.
                replace CRP_FI=1 if SeμCRP_NT4BLM>=5 & SeμCRP_NT4BLM <.
                replace CRP_FI=0 if SeμCRP_NT4BLM<5
tab CRP_FI, missing



*5)HEMOGLOBIN*
gen Hemoglobin_FI=.
*women*
                replace Hemoglobin_FI=1 if BloHb_NT4BLM <11.7 & Sex==0
                replace Hemoglobin_FI=1 if BloHb_NT4BLM >15.3 & BloHb_NT4BLM<. & Sex==0
                replace Hemoglobin_FI=0 if BloHb_NT4BLM >=11.7 & BloHb_NT4BLM<=15.3 & Sex==0
*men*
                replace Hemoglobin_FI=1 if BloHb_NT4BLM <13.4 & Sex==1
                replace Hemoglobin_FI=1 if BloHb_NT4BLM >17.0 & BloHb_NT4BLM<. & Sex==1
                replace Hemoglobin_FI=0 if BloHb_NT4BLM >=13.4 & BloHb_NT4BLM<=17.0 & Sex==1
tab Hemoglobin_FI, missing


*6)CREATININ*
gen Creatinin_FI=.
*women*
                replace Creatinin_FI=1 if SeCrea_NT4BLM <45 & Sex==0
                replace Creatinin_FI=1 if SeCrea_NT4BLM>90 & SeCrea_NT4BLM<. & Sex==0
```

```
            replace Creatinin_FI=0 if SeCrea_NT4BLM>=45 & SeCrea_NT4BLM<=90 & Sex==0
*men*
            replace Creatinin_FI=1 if SeCrea_NT4BLM <60 & Sex==1
            replace Creatinin_FI=1 if SeCrea_NT4BLM >105 & SeCrea_NT4BLM<. & Sex==1
            replace Creatinin_FI=0 if SeCrea_NT4BLM >=60 & SeCrea_NT4BLM<=105 & Sex==1
tab Creatinin_FI, missing


*7) THYROID-STIMULATING HORMONE (TSH)*
gen TSH_FI=.
            replace TSH_FI=1 if SeTSH_NT4BLM<0.5
            replace TSH_FI=1 if SeTSH_NT4BLM>3.6 & SeTSH_NT4BLM<.
            replace TSH_FI=0 if inrange(SeTSH_NT4BLM,0.5,3.6)
tab TSH_FI, missing


*8) LEUKOCYTES*
gen Leukocytes_FI=.
      replace Leukocytes_FI=1 if BloWbc_NT4BLM<4.1
      replace Leukocytes_FI=1 if BloWbc_NT4BLM>9.8 & BloWbc_NT4BLM<.
      replace Leukocytes_FI=0 if inrange(BloWbc_NT4BLM,4.1,9.8)
tab Leukocytes_FI, missing


*9)SERUM CHOLESTEROL*
gen Cholesterol_FI=.
      replace Cholesterol_FI=1 if SeChol_NT4BLM<3.9
      replace Cholesterol_FI=1 if SeChol_NT4BLM>7.8 & SeChol_NT4BLM<.
      replace Cholesterol_FI=0 if inrange(SeChol_NT4BLM,3.9,7.8)
tab Cholesterol_FI, missing


*10)GRANULOCYTES DISTRIBUTION %*
gen Granulocytes_FI=.
      replace Granulocytes_FI=1 if BloNc_NT4BLM<1.8
      replace Granulocytes_FI=1 if BloNc_NT4BLM>6.9 & BloNc_NT4BLM<.
      replace Granulocytes_FI=0 if inrange(BloNc_NT4BLM,1.8,6.9)
tab Granulocytes_FI, missing


*11)TRIGLYCERIDER*
gen Triglycerider_FI=.
      replace Triglycerider_FI=1 if SeTrig_NT4BLM<0.45
      replace Triglycerider_FI=1 if SeTrig_NT4BLM>2.6 & SeTrig_NT4BLM<.
      replace Triglycerider_FI=0 if inrange(SeTrig_NT4BLM,0.45,2.6)
tab Triglycerider_FI, missing

*----------------------------EXAMINE-FI ,ITEM 12-25-----------------------------*

*12) GRIP STRENGTH*
gen GripStrength_FI=.
*women*
            replace GripStrength_FI=1 if GripStre_NT4Eld<16 & Sex==0
            replace GripStrength_FI=0.5 if inrange(GripStre_NT4Eld,16,19.9) & Sex==0
            replace GripStrength_FI=0 if GripStre_NT4Eld >=20 & GripStre_NT4Eld<. & Sex==0
```

```
*men*
        replace GripStrength_FI=1 if GripStre_NT4Eld<26 & Sex==1
        replace GripStrength_FI=0.5 if inrange(GripStre_NT4Eld,26,31.9) & Sex==1
        replace GripStrength_FI=0 if GripStre_NT4Eld >=32 & GripStre_NT4Eld<. & Sex==1
tab GripStrength_FI, missing


*13) GAIT SPEED 4-M USUAL PACE*
*Participants registering as "Exempted, unable to stand" during height/weight measurement
(HeiWeiMea_NT4Eld) or responding "No, I can not walk" on the walking aids question (WalkAid_NT4BLQ2),
and having missing on the gait speed item (GaitSpeed_FI) set to score 1.*
gen GaitSpeed_FI=.
        replace GaitSpeed_FI=1 if SppbGait_NT4Eld==0
        replace GaitSpeed_FI=0.75 if SppbGait_NT4Eld==1
        replace GaitSpeed_FI=0.5 if SppbGait_NT4Eld==2
        replace GaitSpeed_FI=0.25 if SppbGait_NT4Eld==3
        replace GaitSpeed_FI=0 if SppbGait_NT4Eld==4
        replace GaitSpeed_FI=1 if GaitNotAbl_NT4Eld==1 & GaitSpeed_FI==.
        replace GaitSpeed_FI=1 if  HeiWeiMea_NT4Eld==3 & GaitSpeed_FI==.
        replace GaitSpeed_FI=1 if WalkAid_NT4BLQ2==3 & GaitSpeed_FI==.
tab GaitSpeed_FI, missing


*14) FIVE-TIMES CHAIR RISE*
*Participants registering as "Exempted, unable to stand" during Height/weight measurement,
(HeiWeiMea_NT4Eld) and having missing on the chair raise item (ChairRise_FI) set to score 1*
gen ChairRise_FI=.
        replace ChairRise_FI=1 if SppbChair_NT4Eld==0
        replace ChairRise_FI=0.75 if SppbChair_NT4Eld==1
        replace ChairRise_FI=0.5 if SppbChair_NT4Eld==2
        replace ChairRise_FI=0.25 if SppbChair_NT4Eld==3
        replace ChairRise_FI=0 if SppbChair_NT4Eld==4
        replace ChairRise_FI=1 if ChairNotAbl_NT4Eld==1 & ChairRise_FI==.
        replace ChairRise_FI=1 if  HeiWeiMea_NT4Eld==3 & ChairRise_FI==.
tab ChairRise_FI, missing


/*15)MOCA LANGUAGE*
Calculated using the three steps below:*/
/*STEP 1:  Combine subscore animal naming, (MocaNamSubS_NT4Eld), (scale 0-3) and subscore verbal
fluncy, (MocaSentSubS_NT4Eld), (scale 0-2) to a new variable named Moca_Language, scale 0-5.*/
summ MocaNamSubS_NT4Eld MocaSentSubS_NT4Eld
egen Moca_Language_validitems=rownonmiss (MocaNamSubS_NT4Eld MocaSentSubS_NT4Eld)
egen Moca_Language = rowtotal(MocaNamSubS_NT4Eld MocaSentSubS_NT4Eld) if
Moca_Language_validitems==2
tab Moca_Language, missing


*STEP 2: Create a dichotomous variable that determines whether or not SIB is measured*
egen SIB_validitems=rownonmiss (SIBLangNam_NT4Eld SIBOrien_NT4Eld SIBLangMo_NT4Eld
SIBMem_NT4Eld SIBAtten_NT4Eld SIBLangFo_NT4Eld SIBLangSpo_NT4Eld SIBSpoDemo_NT4Eld)
tab SIB_validitems, missing

/*STEP 3: Create a dichotomous variable named Moca_Lang_FI to HUNT-FIScore 5= 0 on FI, Score 3-4= 0,5
på FI, and score <=2, or completed Severe Impairment Battery (SIB) instead of MoCA=1 on FI*/
recode Moca_Language (0=1)(1 2=1)(3 4 =0.5) (5=0), gen(Moca_Lang_FI)
    replace Moca_Lang_FI=1 if inrange(SIB_validitems, 1,8) & Moca_Lang_FI==.
```

tab Moca_Lang_FI, missing

/*16) MOCA ATTENTION*
Calculated using the two steps below:*/
/*STEP 1: Combine subscore series of numbers,(MocaDigSpaSubS_NT4Eld) (scale 0-2). subscore series of letters, (MocaLetTapSubS_NT4Eld), (scale 0-1) and subscore serial subtraction, (Moca7SubtrSumS_NT4Eld) (scale 0-3) to a new variable named Moca_Attention, scale 0-6. Score 5-6= 0 on FI, Score 2-4= 0,5 on FI, score <= 1, or completed Severe Impairment Battery (SIB) instead of MoCA=1 on FI*/
egen Moca_Attention_validitems=rownonmiss (MocaDigSpaSubS_NT4Eld MocaLetTapSubS_NT4Eld Moca7SubtrSumS_NT4Eld)
egen Moca_Attention = rowtotal(MocaDigSpaSubS_NT4Eld MocaLetTapSubS_NT4Eld Moca7SubtrSumS_NT4Eld) if Moca_Attention_validitems==3
tab Moca_Attention, missing

* STEP 2: Create a dichotomous variable Moca_Attention_FI to Frailty Index*
recode Moca_Attention(0 1=1)(2 3 4=0.5)(5 6 =0), gen(Moca_Attention_FI)
replace Moca_Attention_FI=1 if inrange(SIB_validitems, 1,8) & Moca_Attention_FI==.
tab Moca_Attention_FI, missing

17) MOCA MEMORY
gen Moca_Memory_FI=.
replace Moca_Memory_FI=1 if MocaMemSubS_NT4Eld==0
replace Moca_Memory_FI=0.5 if inrange(MocaMemSubS_NT4Eld,1,3) & Moca_Memory_FI==.
replace Moca_Memory_FI=0 if inrange(MocaMemSubS_NT4Eld,4,5) & Moca_Memory_FI==.
replace Moca_Memory_FI=1 if inrange(SIB_validitems, 1,8) & Moca_Memory_FI==.
tab Moca_Memory_FI,missing

18) MOCA ORIENTATION
gen Moca_Orientation_FI=.
replace Moca_Orientation_FI=1 if inrange(MocaOrienSubS_NT4Eld,0,3)
replace Moca_Orientation_FI=1 if inrange(SIB_validitems, 1,8) & Moca_Orientation_FI==.
replace Moca_Orientation_FI=0.5 if inrange(MocaOrienSubS_NT4Eld,4,5) & Moca_Orientation_FI==.
replace Moca_Orientation_FI=0 if MocaOrienSubS_NT4Eld==6 & Moca_Orientation_FI==.
tab Moca_Orientation_FI, missing

19) MOCA EXECUTIVE
gen Moca_Executive_FI=.
replace Moca_Executive_FI=1 if inrange(MocaExecSubS_NT4Eld,0,2)
replace Moca_Executive_FI=1 if inrange(SIB_validitems, 1,8) & Moca_Executive_FI==.
replace Moca_Executive_FI=0.5 if MocaExecSubS_NT4Eld==3 & Moca_Executive_FI==.
replace Moca_Executive_FI=0 if inrange(MocaExecSubS_NT4Eld,4,5)
tab Moca_Executive_FI, missing

20) SYSTOLIC BLOOD PRESSURE
gen Syst_BP_FI=.
replace Syst_BP_FI=1 if BPSystMn23_NT4BLM >140 & BPSystMn23_NT4BLM<.
replace Syst_BP_FI=1 if BPSystMn23_NT4BLM <90 & BPSystMn23_NT4BLM<.
replace Syst_BP_FI=0 if inrange(BPSystMn23_NT4BLM,90,140)
tab Syst_BP_FI, missing

```
*21) DIASTOLIC BLOOD PRESSURE*
gen Dia_BP_FI=.
	replace Dia_BP_FI=1 if BPDiasMn23_NT4BLM>90 & BPDiasMn23_NT4BLM<.
	replace Dia_BP_FI=1 if BPDiasMn23_NT4BLM<60 & BPDiasMn23_NT4BLM<.
	replace Dia_BP_FI=0 if inrange(BPDiasMn23_NT4BLM,60,90)
tab Dia_BP_FI, missing

*22) BODY MASS INDEX*
gen BMI_FI=.
	replace BMI_FI = 1 if Bmi_NT4BLM < 22 & Bmi_NT4BLM != .
	replace BMI_FI = 0.5 if Bmi_NT4BLM >= 30 & Bmi_NT4BLM != .
	replace BMI_FI = 0 if Bmi_NT4BLM >= 22 & Bmi_NT4BLM < 30 & Bmi_NT4BLM != .
tab BMI_FI, missing


*23) PULSE*
*Pre-step: calculate mean of 2. and 3. measurement*
*Community-dwelling participants*
gen Pulse_2and3_home= Puls2_NT4BLM+ Puls3_NT4BLM
gen Pulse_Mean_Home= Pulse_2and3_home/2
rename Pulse_Mean_Home Pulse_Home_FI
summ Pulse_Home_FI

*Pre-step: calculate mean of 2. and 3. measurement*
*Nursing home participants*
gen Pulse_2and3_inst= Puls2_NT4EldMIX+ Puls3_NT4EldMIX
gen Pulse_Mean_Inst= Pulse_2and3_inst/2
rename Pulse_Mean_Inst Pulse_Institution_FI
summ Pulse_Institution_FI

gen Pulse_FI=.
	replace Pulse_FI=1 if Pulse_Home_FI >99 & Pulse_Home_FI<.
	replace Pulse_FI=1 if Pulse_Institution_FI>99 & Pulse_Institution_FI<.
	replace Pulse_FI=1 if Pulse_Home_FI<60 & Pulse_FI==.
	replace Pulse_FI=1 if Pulse_Institution_FI <60 & Pulse_FI==.
	replace Pulse_FI=0 if inrange(Pulse_Home_FI,60,99) & Pulse_FI==.
	replace Pulse_FI=0 if inrange(Pulse_Institution_FI,60,99) & Pulse_FI==.
tab Pulse_FI, missing


*24) OXYGEN SATURATION*
*Pre-step: calculate mean of 2. and 3. measurement*
*Community-dwelling participants*
generate double O2_2and3_home= O2_NT4BLM+ O3_NT4BLM
generate O2_Mean_Home= O2_2and3_home/2
rename O2_Mean_Home O2_Home_FI
summ O2_Home_FI

*Pre-step: calculate mean of 2. and 3. measurement*
*Nursing home particpants*
generate double O2_2and3_inst= O2_NT4EldMIX+ O3_NT4EldMIX
generate O2_Mean_Inst= O2_2and3_inst/2
rename O2_Mean_Inst O2_Institution_FI
summ O2_Institution_FI
```

```
gen SpO2_FI=.
	replace SpO2_FI=1 if O2_Home_FI<96
	replace SpO2_FI=1 if O2_Institution_FI<96 & SpO2_FI==.
	replace SpO2_FI=0 if O2_Home_FI>=96 & O2_Home_FI<.
	replace SpO2_FI=0 if O2_Institution_FI>=96 & O2_Institution_FI<.
tab SpO2_FI, missing


*25) DEMENTIA DIAGNOSED BY MEDICAL DOCTOR*
recode DiagDem_NT4Eld (0=0 "No") (1 2 3 4 5 6 7=1 "Yes") (9=.), gen(Dementia_FI)
label variable Dementia_FI "Dementia diagnosed by medical doctor"
tab Dementia_FI, missing

*---------------------------SELF-REPORT-FI, ITEM 26-35------------------------------*

*26) SELF-REPORTED HEALTH*
recode Healt_NT4BLQ1 (3 4=0) (1 2=1), gen(SelfHealth_FI)
label variable SelfHealth_FI "How is your health at the moment?"
tab SelfHealth_FI, missing


*27) DEPRESSION*
*Calculated using the four steps below, to combine variables from CONOR/NPI and HADS:*
* STEP 1: Dichotomize the Conor-variable (FeelDeprL2W_NT4BLQ1), primary outcome for community-
dwelling participants:*
codebook FeelDeprL2W_NT4BLQ1
recode FeelDeprL2W_NT4BLQ1 (1=0 "No") (2/4=1 "Yes"), generate (Conor_Depression_FI)
label values Conor_Depression_FI dichotomous
tab Conor_Depression_FI, missing
codebook Conor_Depression_FI


/*STEP 2: Dichotomize the NPI-variable (NPINHDepr_NT4EldMI), primary outcome for participants living in
nursing homes. Participants registering with "not relevant" are coded as missing*/
codebook NPINHDepr_NT4EldMI
recode NPINHDepr_NT4EldMI (0=0 "No")(1=1 "Yes") (2=.), generate (NPI_Depression_FI)
label values NPI_Depression_FI dichotomous
tab NPI_Depression_FI, missing
codebook NPI_Depression_FI

/*STEP 3: Dichotomize secondary choice of outcome; total score HADS Depression (HADSDepr_NT4BLQ2) if
CONOR/NPI is missing*/
generate HADS_Depr_Dichotomous=0
	replace HADS_Depr_Dichotomous=1 if HADSDepr_NT4BLQ2==8
	replace HADS_Depr_Dichotomous=1 if HADSDepr_NT4BLQ2 >8 & HADSDepr_NT4BLQ2<.
	replace HADS_Depr_Dichotomous=. if HADSDepr_NT4BLQ2==.
tab HADS_Depr_Dichotomous, missing

*STEP 4: Combine CONOR, NPI and  HADS to final variable Depression_FI*
generate Depression_FI=1 if NPI_Depression_FI==1
	replace Depression_FI=1 if Conor_Depression_FI==1 & Depression_FI==.
	replace Depression_FI=1 if HADS_Depr_Dichotomous==1 & Depression_FI==.
	replace Depression_FI=0 if NPI_Depression_FI==0 & Depression_FI==.
	replace Depression_FI=0 if Conor_Depression_FI==0 & Depression_FI==.
	replace Depression_FI=0 if HADS_Depr_Dichotomous==0 & Depression_FI==.
tab Depression_FI, missing


*28) ANXIETY*
```

Calculated using the four steps below, to combine variables from CONOR/NPI and HADS:
/* STEP 1: Dichotomize the Conor-variable (FeelAnxiL2W_NT4BLQ1), primary outcome for community-dwelling participants:*/
codebook FeelAnxiL2W_NT4BLQ1
recode FeelAnxiL2W_NT4BLQ1 (1=0 "No") (2/4=1 "Yes"), generate (Conor_Anxiety_FI)
label values Conor_Anxiety_FI dichotomous
tab Conor_Anxiety_FI
codebook Conor_Anxiety_FI

STEP 2: Dichotomize the NPI-variable (NPINHAnxi_NT4EldMI), primary outcome for participants living in nursing homes. Participants registering with "not relevant" are coded as missing
codebook NPINHAnxi_NT4EldMI
recode NPINHAnxi_NT4EldMI (0=0 "No")(1=1 "Yes") (2=.), generate (NPI_Anxiety_FI)
tab NPI_Anxiety_FI
codebook NPI_Anxiety_FI

STEP 3: Dichotomize secondary choice of outcome; total score HADS Anxiety (HADSAnxi_NT4BLQ2) if CONOR/NPI is missing
generate HADS_Anxiety_Dichotomous=0
    replace HADS_Anxiety_Dichotomous=1 if HADSAnxi_NT4BLQ2==8
    replace HADS_Anxiety_Dichotomous=1 if HADSAnxi_NT4BLQ2 >8
    replace HADS_Anxiety_Dichotomous=. if HADSAnxi_NT4BLQ2==.
tab HADS_Anxiety_Dichotomous, missing

STEP 4: Combine CONOR, NPI and HADS to final variable Anxiety_FI
generate Anxiety_FI=1 if NPI_Anxiety_FI==1
    replace Anxiety_FI=1 if Conor_Anxiety_FI==1 & Anxiety_FI==.
    replace Anxiety_FI=1 if HADS_Anxiety_Dichotomous==1 & Anxiety_FI==.
    replace Anxiety_FI=0 if NPI_Anxiety_FI==0 & Anxiety_FI==.
    replace Anxiety_FI=0 if Conor_Anxiety_FI==0 & Anxiety_FI==.
    replace Anxiety_FI=0 if HADS_Anxiety_Dichotomous==0 & Anxiety_FI==.
tab Anxiety_FI, missing

29)EXHAUSTION/TIREDNESS CURRENT
Primary variable: TireCu_NT4BLQ2. When TireCu_NT4BLQ2 is missing, use secondary choice variable FeelStro_NT4BLQ1, which need to be dichotomized to the variable Conor_strong, before combining to the final variabel Tired_FI. Use the two steps below:
STEP 1: Dichotomize Conor FeelStro_NT4BLQ1
recode FeelStro_NT4BLQ1 (7=0)(6=0)(5=0)(4=1)(3=1)(2=1)(1=1), generate (Conor_Strong)

STEP 2: Combine to create final variable Tired_FI.
generate Tired_FI=1 if TireCu_NT4BLQ2==1
    replace Tired_FI=1 if Conor_Strong==0 & Tired_FI==.
    replace Tired_FI=0 if TireCu_NT4BLQ2==0 & Tired_FI==.
    replace Tired_FI=0 if Conor_Strong==1 & Tired_FI==.
tab Tired_FI, missing

30) BODYPAIN
Combine variable on physical pain (MSPaChrL4W_NT4BLQ1) and joint pain (JoPaMor6WLY_NT4BLQ1)

Preparation, dichotomize variable MSPaChrL4W_NT4BLQ1:
recode MSPaChrL4W_NT4BLQ1 (1 2 3=0) (4 5 6=1), gen(Physical_Pain_FI)

```
generate BodyPain_FI=.
	replace BodyPain_FI=1 if JoPaMor6WLY_NT4BLQ1==1
	replace BodyPain_FI=1 if Physical_Pain_FI==1 & BodyPain_FI==.
	replace BodyPain_FI=0 if JoPaMor6WLY_NT4BLQ1==0 & BodyPain_FI==.
	replace BodyPain_FI=0 if Physical_Pain_FI==0 & BodyPain_FI==.
	replace BodyPain_FI=. if JoPaMor6WLY_NT4BLQ1==. & Physical_Pain_FI==.
tab BodyPain_FI, missing


*31) TOOTHPAIN*
generate ToothPain_FI=.
	replace ToothPain_FI=1 if PaTootMouL4W_NT4EldMI==1
	replace ToothPain_FI=0 if PaTootMouL4W_NT4EldMI==0
tab ToothPain_FI, missing

*32)LEVEL OF PHYSICAL ACTIVITY*
/*Preparation: dichotomize variables for community-dwelling participants (ExeF_NT4BLQ1) and nursing home participants (PADLPhyMov_NT4Eld)*/
codebook ExeF_NT4BLQ1
recode ExeF_NT4BLQ1 (1/2=1)(3/5=0) , generate (PhysAct_home)

codebook PADLPhyMov_NT4Eld
recode PADLPhyMov_NT4Eld (1/2=0)(3/5=1) , generate (PhysAct_nursinghomes)

*Combine to final variable PhysAct_FI*
gen PhysAct_FI=0
replace PhysAct_FI=1 if PhysAct_home==1
replace PhysAct_FI=1 if PhysAct_nursinghomes==1
replace PhysAct_FI=. if PhysAct_home==. & PhysAct_nursinghomes==.
tab PhysAct_FI, missing


*33) LOSS OF APPETITE*
generate Appetite_FI=.
	replace Appetite_FI=1 if PooAppeL4W_NT4EldMI==1
	replace Appetite_FI=0 if PooAppeL4W_NT4EldMI==0
tab Appetite_FI, missing


*34) CHEW FUNCTION*
generate Chewing_FI=.
	replace Chewing_FI=0 if ChewFo_NT4EldMI==1
	replace Chewing_FI=1 if ChewFo_NT4EldMI==0
tab Chewing_FI, missing


*35)INSOMNIA*
*Calculated using the two steps below:*
*STEP 1: Dichotomize and combine the three variables "difficulty falling asleep"(InsomEvnL3M_NT4BLQ1), "repeatedly wakening up during the night" (InsomNigL3M_NT4BLQ1) and "wake up too early"(InsomMornL3M_NT4BLQ1) (Våkner tidlig om morgenen) used in the sample of community-dwelling participants*
recode InsomEvnL3M_NT4BLQ1 (1=0) (2=0) (3=1), generate (Insom_evening_home)
recode InsomNigL3M_NT4BLQ1 (1=0) (2=0) (3=1), generate (Insom_night_home)
recode InsomMornL3M_NT4BLQ1 (1=0) (2=0) (3=1), generate (Insom_morning_home)
```

```
*STEP 2- Create the final variable Insomnia_FI, and include the variable NPINHSle_NT4EldMI used in the nursing home sample for å dekke beboere på sykehjem.*
generate Insomnia_FI=1 if Insom_evening_home==1
	replace Insomnia_FI=1 if Insom_night_home==1 & Insomnia_FI==.
	replace Insomnia_FI=1 if Insom_morning_home==1 & Insomnia_FI==.
	replace Insomnia_FI=1 if NPINHSle_NT4EldMI==1 & Insomnia_FI==.
	replace Insomnia_FI=0 if Insom_night_home==0 & Insomnia_FI==.
	replace Insomnia_FI=0 if Insom_morning_home==0 & Insomnia_FI==.
	replace Insomnia_FI=0 if NPINHSle_NT4EldMI==0 & Insomnia_FI==.
	replace Insomnia_FI=0 if Insom_evening_home==0 & Insomnia_FI==.
tab Insomnia_FI, missing
```

---CALCULATE FI_SCORES_---

```
* Estimate Valid number of FI-deficits.Participants must have 80% (min 28 items) in order to calculate score*

egen FI_35_validitems=rownonmiss (Albumin_FI HbA1c_FI GFR_FI CRP_FI Hemoglobin_FI Creatinin_FI TSH_FI Leukocytes_FI Cholesterol_FI Granulocytes_FI Triglycerider_FI GripStrength_FI GaitSpeed_FI ChairRise_FI Moca_Lang_FI Moca_Attention_FI Moca_Memory_FI Moca_Orientation_FI Moca_Executive_FI Syst_BP_FI Dia_BP_FI BMI_FI Pulse_FI SpO2_FI Dementia_FI SelfHealth_FI Depression_FI Anxiety_FI Tired_FI BodyPain_FI ToothPain_FI PhysAct_FI Appetite_FI Chewing_FI Insomnia_FI )

tab FI_35_validitems, missing

*Select participants with >=80% valid items for inlusion in analyses*
gen FI_35_included=.
	replace FI_35_included=1 if inrange(FI_35_validitems,28,35)
	replace FI_35_included=0 if FI_35_validitems <28
label define Analysis 0"Excluded" 1"Included"
label values FI_35_included "Analysis"
label variable FI_35_included "Participants with >=80% valid items"

*Estimate FI_35_score*

egen FI_35_score=rmean(Albumin_FI HbA1c_FI GFR_FI CRP_FI Hemoglobin_FI Creatinin_FI TSH_FI Leukocytes_FI Cholesterol_FI Granulocytes_FI Triglycerider_FI GripStrength_FI GaitSpeed_FI ChairRise_FI Moca_Lang_FI Moca_Attention_FI Moca_Memory_FI Moca_Orientation_FI Moca_Executive_FI Syst_BP_FI Dia_BP_FI BMI_FI Pulse_FI SpO2_FI Dementia_FI SelfHealth_FI Depression_FI Anxiety_FI Tired_FI BodyPain_FI ToothPain_FI PhysAct_FI Appetite_FI Chewing_FI Insomnia_FI) if FI_35_included==1
summ FI_35_score
hist FI_35_score

*Categorize frailty*

*Cut-offs for presentation in Prevalence paper:  Robust: <0.15, Prefrail: 0.15-0.24, Frail:>=0.25*

gen Frail_byFI_35=.
	replace Frail_byFI_35=0 if FI_35_score<0.15
	replace Frail_byFI_35=1 if FI_35_score>=0.15 &FI_35_score<0.25
	replace Frail_byFI_35=2 if FI_35_score>=0.25 & FI_35_score<.
label define frail 0"Robust" 1"Prefrail" 2"Frail"
label values Frail_byFI frail
tab Frail_byFI
```

Stata syntax Fried criteria

```
*1) EXHAUSTION/TIREDNESS CURRENT*
*Primary variable: TireCu_NT4BLQ2. When TireCu_NT4BLQ2 is missing, use secondary choice variable FeelStro_NT4BLQ1, which need to be dichotomized to the variable Conor_strong, before combining to the final variabel Fried_tired. Use the two steps below:*
*STEP 1: Dichotomize Conor FeelStro_NT4BLQ1*
recode FeelStro_NT4BLQ1 (7=0)(6=0)(5=0)(4=1)(3=1)(2=1)(1=1), generate (Conor_Strong)

*STEP 2: Combine to create final variable Fried_tired.*
generate Fried_tired=1 if TireCu_NT4BLQ2==1
        replace Fried_tired=1 if Conor_Strong==0 & Fried_tired==.
        replace Fried_tired=0 if TireCu_NT4BLQ2==0 & Fried_tired==.
        replace Fried_tired=0 if Conor_Strong==1 & Fried_tired==.
tab Fried_tired, missing



*2) GRIP STRENGTH*
*Sex: Women=0, men=1*
gen Fried_grip=0
        replace Fried_grip=1 if Sex==1 & Bmi_NT4BLM<=24 & GripStre_NT4Eld<29
        replace Fried_grip=1 if Sex==1 & Bmi_NT4BLM>24 & Bmi_NT4BLM<=26 & GripStre_NT4Eld<30
        replace Fried_grip=1 if Sex==1 & Bmi_NT4BLM>26 & Bmi_NT4BLM<=28 & GripStre_NT4Eld<30
        replace Fried_grip=1 if Sex==1 & Bmi_NT4BLM>28 & Bmi_NT4BLM<. & GripStre_NT4Eld<32
        replace Fried_grip=1 if Sex==0 & Bmi_NT4BLM<=23 & GripStre_NT4Eld<17
        replace Fried_grip=1 if Sex==0 & Bmi_NT4BLM>23 & Bmi_NT4BLM<=26 &
        GripStre_NT4Eld<17.3
        replace Fried_grip=1 if Sex==0 & Bmi_NT4BLM>26 & Bmi_NT4BLM<=29 & GripStre_NT4Eld<18
        replace Fried_grip=1 if Sex==0 & Bmi_NT4BLM>29 & Bmi_NT4BLM<. & GripStre_NT4Eld<21

        replace Fried_grip=. if GripStre_NT4Eld==.
        replace Fried_grip=1 if GripStreLeNotAbl_NT4Eld==1 & GripStreRiNotAbl_NT4Eld==1 &
        GripStre_NT4Eld==.
tab Fried_grip, missing


*3) GAIT SPEED*
*There are two different variables measuring height in the sample:
*Hei_NT4Eld - nursing home*
*Hei_NT4BLM - community-dwelling*

*Participants registering as "Exempted, unable to stand" during height/weight measurement (HeiWeiMea_NT4Eld) or responding "No, I can not walk" on the walking aids question (WalkAid_NT4BLQ2), and having missing on the gait speed item (Fried_gait) set to score one.*

gen Fried_gait=0
        replace Fried_gait=1 if Sex==1 & Hei_NT4BLM<=173 & GaitSpe_NT4Eld<=0.6531
        replace Fried_gait=1 if Sex==1 & Hei_NT4Eld<=173 & GaitSpe_NT4Eld<=0.6531 &
        Hei_NT4BLM==.
        replace Fried_gait=1 if Sex==1 & Hei_NT4BLM>173  & Hei_NT4BLM<. & GaitSpe_NT4Eld<=0.762
        replace Fried_gait=1 if Sex==1 & Hei_NT4Eld>173  & Hei_NT4Eld<. & GaitSpe_NT4Eld<=0.762 &
        Hei_NT4BLM==.
        replace Fried_gait=1 if Sex==0 & Hei_NT4BLM<=159 & GaitSpe_NT4Eld<=0.6531
        replace Fried_gait=1 if Sex==0 & Hei_NT4Eld<=159 & GaitSpe_NT4Eld<=0.6531 &
        Hei_NT4BLM==.
        replace Fried_gait=1 if Sex==0 & Hei_NT4BLM>159  & Hei_NT4BLM<. & GaitSpe_NT4Eld<=0.762
        replace Fried_gait=1 if Sex==0 & Hei_NT4Eld>159  & Hei_NT4Eld<. & GaitSpe_NT4Eld<=0.762 &
        Hei_NT4BLM==.
        replace Fried_gait=. if GaitSpe_NT4Eld==.
```

```
        replace Fried_gait=1 if GaitNotAbl_NT4Eld==1 & GaitSpe_NT4Eld==.
        replace Fried_gait=1 if HeiWeiMea_NT4Eld==3 & Fried_gait==.
        replace Fried_gait=1 if WalkAid_NT4BLQ2==3 & Fried_gait==.
tab Fried_gait, missing


*4) WEIGHT LOSS*
gen Fried_weight=0
        replace Fried_weight=1 if WeiRedL6M_NT4BLQ1==1
        replace Fried_weight=1 if Bmi_NT4BLM<22 & WeiRedL6M_NT4BLQ1==.
        replace Fried_weight=. if Bmi_NT4BLM==. & WeiRedL6M_NT4BLQ1==.
tab Fried_weight, missing


*5) PHYSICAL ACTIVITY LEVEL*
*Preparation: dichotomize variables for community-dwelling participants (ExeF_NT4BLQ1) and nursing home participants (PADLPhyMov_NT4Eld)*
codebook ExeF_NT4BLQ1
recode ExeF_NT4BLQ1 (1/2=1)(3/5=0) , generate (PhysAct_home)

codebook PADLPhyMov_NT4Eld
recode PADLPhyMov_NT4Eld (1/2=0)(3/5=1) , generate (PhysAct_nursinghomes)

*Participants registering as "Exempted, unable to stand" during height/weight measurement (HeiWeiMea_NT4Eld) or responding "No, I can not walk" on the walking aids question (WalkAid_NT4BLQ2), and having missing on the gait speed item (GaitSpeed_FI) set to score one.*

*Combine to final variable Fried_PhysAct*
gen Fried_physact=0
replace Fried_physact=1 if PhysAct_home==1
replace Fried_physact=1 if PhysAct_nursinghomes==1
replace Fried_physact=. if PhysAct_home==. & PhysAct_nursinghomes==.
replace Fried_physact=1 if HeiWeiMea_NT4Eld==3 & Fried_physact==.
replace Fried_physact=1 if WalkAid_NT4BLQ2==3 & Fried_physact==.
tab Fried_physact, missing


*CALCULATE FRIED SCORE*

*Estimate valid number of Fried items. Participants must have 80% (min 4 items) in order to be included in final analyses*

egen Fried_Valid_Items=rownonmiss(Fried_tired Fried_grip Fried_gait Fried_weight Fried_physact)
tab Fried_Valid_Items

*Select participants with >=80% valid items for inclusion in analyses*
gen Fried_included=0
        replace Fried_included=1 if Fried_Valid_Items >=4
label define Analyse 0"Excluded from analyses" 1"Included in analyses"
label values Fried_included Analyse
label variable Fried_included "Participants with >=4 valid items on Fried criteria"
tab Fried_included

*Categorize frailty*
summ Fried_tired Fried_grip Fried_gait Fried_weight Fried_physact
egen Fried_sum = rowtotal(Fried_tired Fried_grip Fried_gait Fried_weight Fried_physact) if Fried_included==1
recode Fried_sum (0=0)(1 2=1)(3 4 5=2), gen(Frail_byFried)
label define frail 0"Robust" 1"Pre-frail" 2"Frail"
label values Frail_byFried frail
tab Frail_byFried
```

References for cut-off values

1. Fried LP, Tangen CM, Walston J et al (2001) Frailty in older adults: evidence for a phenotype. J Gerontol A Biol Sci Med Sci 56:M146–156. https://doi.org/10.1093/gerona/56.3.M146
2. Cederholm T, Jensen GL, Correia M et al (2019) GLIM criteria for the diagnosis of malnutrition - A consensus report from the global clinical nutrition community. Clin Nutr 38:1–9. https://doi.org/10.1016/j.clnu.2018.08.002
3. Rockwood K, Mitnitski A (2007) Frailty in relation to the accumulation of deficits. J Gerontol A Biol Sci Med Sci 62:722–727. https://doi.org/10.1093/gerona/62.7.722
4. Theou O, Haviva C, Wallace L, Searle SD, Rockwood K (2023) How to construct a frailty index from an existing dataset in 10 steps. Age Ageing 52. https://doi.org/10.1093/ageing/afad221.
5. Searle SD, Mitnitski A, Gahbauer EA, Gill TM, Rockwood K (2008) A standard procedure for creating a frailty index. BMC Geriatr 8:24. https://doi:10.1186/1471-2318-8-24.
6. Kim DH, Afilalo J, Shi SM et al (2019) Evaluation of changes in functional status in the year after aortic valve replacement. JAMA Intern Med 179:383–391. https://doi.org/10.1001/jamainternmed.2018.6738
7. Guralnik JM, Simonsick EM, Ferrucci L et al (1994) A short physical performance battery assessing lower extremity function: association with self-reported disability and prediction of mortality and nursing home admission. J Gerontol 49: M85–94.https://doi.org/10.1093/geronj/49.2.m85
8. Jayanama K, Theou O, Godin J et al (2022) Relationship of body mass index with frailty and all-cause mortality among middle-aged and older adults. BMC Med 20:404. https://doi:10.1186/s12916-022-02596-7.
9. Vold ML, Aasebo U, Wilsgaard T, Melbye H (2015) Low oxygen saturation and mortality in an adult cohort: the Tromso study. BMC Pulm Med 15:9. https://doi:10.1186/s12890-015-0003-5.
10. Stordal E, Bjartveit KM, Dahl NH, Krüger O, Mykletun A, Dahl AA (2001) Depression in relation to age and gender in the general population: The Nord-Trøndelag Health Study (HUNT). Acta Psychiatr Scand 104:210–6. https://doi.org/10.1034/j.1600-0447.2001.00130.x
11. Song X, Mitnitski A, Rockwood K (2010) Prevalence and 10-year outcomes of frailty in older adults in relation to deficit accumulation. J Am Geriatr Soc 58:681–687. https://doi.org/10.1111/j.1532-5415.2010.02764.x
12. Kim DH, Glynn RJ, Avorn J et al (2019) Validation of a claims-based frailty index against physical performance and adverse health outcomes in The Health and Retirement Study. J Gerontol A Biol Sci Med Sci 74: 1271–1276. https://doi.org/10.1093/gerona/gly197
13. Shi SM, McCarthy EP, Mitchell S, Kim DH (2020) Changes in predictive performance of a frailty index with availability of clinical domains. J Am Geriatr Soc 68:1771–1777. https://doi.org/10.1111/jgs.16436
14. Helse Nord-Trøndelag HF N-THT (2021) ALM Referanseverdier for voksne ≥18 år, 2021, versjon 1.13. [ALM, reference values for adults ≥ 18 years, 2021, version 1.13]. Håndbok til tolkning av laboratorieprøver [Handbook for interpretation of laboratory tests]: Avdeling for Laboratoriemedisin. Helse Nord-Trøndelag HF [Department of Laboratory Medicine, Nord-Trøndelag Hospital Trust]
